# Achieving Congestion Mitigation Using Distributed Power Control for Spectrum Sensor Nodes in Sensor Network-Aided Cognitive Radio Ad Hoc Networks

**DOI:** 10.3390/s17092132

**Published:** 2017-09-15

**Authors:** Long Zhang, Haitao Xu, Fan Zhuo, Hucai Duan

**Affiliations:** 1 School of Information and Electrical Engineering, Hebei University of Engineering, Handan 056038, China; fnzhuo@outlook.com; 2 School of Computer and Communication Engineering, University of Science and Technology Beijing, Beijing 100083, China; xuhaitao@ustb.edu.cn; 3 China Telecommunication Technology Labs-Terminals, China Academy of Information and Communications Technology, Beijing 100191, China; duanhucai@caict.ac.cn

**Keywords:** cognitive radio, sensor network, congestion mitigation, power control

## Abstract

The data sequence of spectrum sensing results injected from dedicated spectrum sensor nodes (SSNs) and the data traffic from upstream secondary users (SUs) lead to unpredictable data loads in a sensor network-aided cognitive radio ad hoc network (SN-CRN). As a result, network congestion may occur at a SU acting as fusion center when the offered data load exceeds its available capacity, which degrades network performance. In this paper, we present an effective approach to mitigate congestion of bottlenecked SUs via a proposed distributed power control framework for SSNs over a rectangular grid based SN-CRN, aiming to balance resource load and avoid excessive congestion. To achieve this goal, a distributed power control framework for SSNs from interior tier (IT) and middle tier (MT) is proposed to achieve the tradeoff between channel capacity and energy consumption. In particular, we firstly devise two pricing factors by considering stability of local spectrum sensing and spectrum sensing quality for SSNs. By the aid of pricing factors, the utility function of this power control problem is formulated by jointly taking into account the revenue of power reduction and the cost of energy consumption for IT or MT SSN. By bearing in mind the utility function maximization and linear differential equation constraint of energy consumption, we further formulate the power control problem as a differential game model under a cooperation or noncooperation scenario, and rigorously obtain the optimal solutions to this game model by employing dynamic programming. Then the congestion mitigation for bottlenecked SUs is derived by alleviating the buffer load over their internal buffers. Simulation results are presented to show the effectiveness of the proposed approach under the rectangular grid based SN-CRN scenario.

## 1. Introduction

Cognitive radio (CR) [1] has newly emerged as a promising solution to improve the spectrum utilization by allowing unlicensed secondary users (SUs) to access the idle licensed spectrum. In a CR network (CRN), SUs can periodically sense the licensed spectrum and opportunistically access the spectrum holes or spectrum opportunities (SOPs) unoccupied by primary users (PUs). Most of the existing research efforts in CRNs mainly focus on the issues of the physical and MAC layers for an infrastructure-based single hop scenario, such as spectrum sensing, spectrum access and sharing techniques [2,3,4]. In addition, SUs can also form a multi-hop ad hoc network without the support of infrastructure. In a cognitive radio ad hoc network (CRANET) [5], SUs can only access the SOPs by seeking to underlay, overlay, or interweave their signals with those of the existing PUs without significantly impacting their communications.

Spectrum sensing is one of the key enabling technologies for the establishment of CRNs, because it constantly allows for the opportunistic identification and use of the SOPs from a licensed primary network without causing harmful interference to the PUs. In order to improve the sensing performance, collaborative spectrum sensing has been proposed as an effective way to reliably detect the activity of PUs by addressing the issues imposed by the hidden PU terminal problem and the wireless channel impairments, such as the heavy shadowing and fading [6,7,8]. In this way, cooperation is achieved by allowing different SUs to collaborate and share their spectrum sensing results (SSR) through a fusion center (FC), which makes a global decision on the occupancy status of the licensed band. However, this centralized FC is not available in decentralized CRANETs. Clearly, each SU under this scenario must perform the distributed collaborative spectrum sensing, which is preferred to the centralized FC scheme because of its scalability, fault tolerance and flexibility [9].

In order to facilitate the spectrum sensing functionality, high sampling rates, high resolution analog to digital converters with large dynamic range, and high speed signal processors are required to be incorporated into an individual SU transceiver [10], which increases hardware cost and power consumption, especially for the double-radio sensing architecture of SU transceiver. An alternative approach is to adopt the cost-effective and dedicated spectrum sensor nodes (SSNs) that perform distributed collaborative spectrum sensing and report SSR to SUs acting as FCs in CRANETs [11]. Technically, s wireless sensor network can be naturally exploited to assist a CRANET by providing SSR about the current spectrum occupancy of PUs in a cooperation fashion. The concept of sensor network embedded into CRANETs has further called sensor network-aided CRANETs (SN-CRNs), which has been considered as one of the most appealing approaches to perform cost-effective spectrum sensing in CR systems [7,11,12].

Similar to most other traditional wireless networks or wireline Internet, network congestion in SN-CRNs will also occur when offered data load that exceed the available capacity of a SU due to buffer overflow caused by the data sequence of the SSR injected from SSNs together with the data traffic from upstream SUs. This therefore leads to energy consumption of SSNs, aggressive retransmission, queuing delay, and blocking of new flows from upstream SUs. Indubitably, a congestion control technique in the transport layer is essential to balance resource loads and avoid excessive congestion. However, the congestion control mechanism for the traditional Transmission Control Protocol (TCP) via the acknowledgement-triggered or window-based methods was initially designed and optimized to perform in reliable wired links with constrained bit error rates and round trip times (RTTs) [13]. A recent study [14] has reported that the performance of HTTP download deteriorates as much as about 40% under the TCP window control in an IEEE P1900.4 based cognitive wireless system by using User Datagram Protocol (UDP) and TCP transport protocols. On the other hand, some other research efforts about congestion control have also been conducted from the perspective of finding methods to modify the TCP protocol, such as TCP monitoring delayed acknowledgment, segment-based selective acknowledgement, TCP adaptive delayed-acknowledgment window, etc. [15], aiming to accommodate the challenging multi-hop wireless environments. Unfortunately, it has been shown that these methods of TCP modification and extension cannot be directly applied into SN-CRNs due to sudden large-scale bandwidth fluctuation, periodic interruption caused by spectrum sensing and channel switching [16].

Recently, there have also been previous works on congestion control for multi-hop CRANETs from a cross-layer design perspective. In [17], an end-to-end congestion control framework was proposed under the constraint of the non-uniform channel availability by taking into account the interactions from the physical layer to the transport layer. In [18], a cross-layer framework for joint optimization of MAC, scheduling, routing and congestion control was presented to maximize the throughput of a set of multi-hop end-to-end packet flows. However, the end-to-end control policy in [17,18] is ill suited for operation over wireless links characterized by higher RTTs. On the contrary, the hop-by-hop control reacts to congestion faster where the rates are adjusted at intermediate SUs by feedback information about the congestion state of congested SUs. A cross-layer framework to jointly achieve both congestion and power control through a non-convex optimization method was proposed in [19]. In [20], an optimization framework achieving tradeoff between energy efficiency and network utility maximization was devised, which can jointly balance interference, collision, and congestion among SUs by adjusting transmit power, persistence probability, together with data rate simultaneously via interaction between MAC and other layers. However, the proposed frameworks in [19,20] are just suited to mitigate the congestion caused by the data traffic from upstream SUs in multi-hop CRANETs, ignoring the impact of the data sequence of the SSR injected from SSNs on the congestion of SUs.

To the best of our knowledge, aside from some studies on congestion control for CRANETs as mentioned before, there is no related work reported in the literature related to congestion control over SN-CRNs. As a result, there is a strong motivation to explore congestion mitigation approach in SN-CRNs. Under this scenario, it is certainly not a surprise that the channel capacity between any SSN and FC is a concave function of the transmit power of this SSN and channel conditions [21]. In principle, effective transmit power control strategies have been widely used to maximize the total system capacity in conventional celluar wireless networks while adapting to the changing channel and interference conditions. Recent research efforts have achieved the capacity and energy efficiency maximization by devising the optimal power allocation on subchannels in two-tier femtocell networks based on orthogonal frequency division multiple access (OFDMA) [22], together with the optimal power control allocation and sensing time optimization in OFDMA cognitive small cell networks [23]. In addition, the transmission rate of this SSN always depends on channel capacity and is also a function of the transmit power according to the Shannon channel theorem. Thus, the congestion at FC can be controlled and mitigated through the transmission rate adjustment with the help of an optimal power allocation policy for this SSN in the physical layer. During a time interval, the amount of bits of the data sequence of SSR transmitted from this SSN to FC also approximately depends on the channel capacity [24]. For this observation it turns out that we can fully achieve the congestion mitigation for FC by reducing the amount of bits of the data sequence of the SSR transmitted from this SSN, aiming to release the capacity of the internal buffer for FC. In this paper, we propose a congestion mitigation approach by constructing a distributed power control framework for SSNs over the rectangular grid based SN-CRN. The main contributions of this paper are summarized as follows:

1. To evaluate the performance of local spectrum sensing, we present the relative divergence between the detection probability and the false alarm probability for each SSN under any uplink channel via the Kullback-Leibler divergence framework. By the aid of mathematical statistics, we obtain the detection probability and false alarm probability distributions for each SSN, and also model the stability metric of local spectrum sensing as the relative divergence by applying the entropy modeling framework.
2. We propose a distributed power control framework for SSNs from the interior tier (IT) and middle tier (MT) perspective in order to achieve the tradeoff between channel capacity and energy consumption. In particular, the power control problem is formulated as a differential game model by taking into account the utility function maximization together with the linear differential equation constraint with respect to energy consumption. We further present the theoretical results of the optimal solutions to this differential game model in a cooperative or noncooperative manner by using dynamic programming.
3. With the help of the proposed distributed power control framework, we attain the congestion mitigation for bottleneck SU by alleviating its buffer load over its internal buffer. We also rigorously analyze the impact of noncooperative and cooperative optimal transmit power for IT and MT SSNs on the internal buffer of bottleneck SU, respectively.

The rest of paper is organized as follows: Section 2 describes the system model. In Section 3, we present the spectrum sensing quality analysis method based on local spectrum sensing by SSNs. In Section 4, we formulate the distributed power control for IT and MT SSNs as a differential game model, and derive the noncooperative and cooperative optimal solutions. The congestion mitigation approach for bottleneck SU is analyzed rigorously in Section 5. Section 6 presents the simulation results. Finally, Section 7 concludes the paper.

## 2. System Model

### 2.1. Primary Network and Cognitive Radio ad hoc Network Model

**Figure 1:**
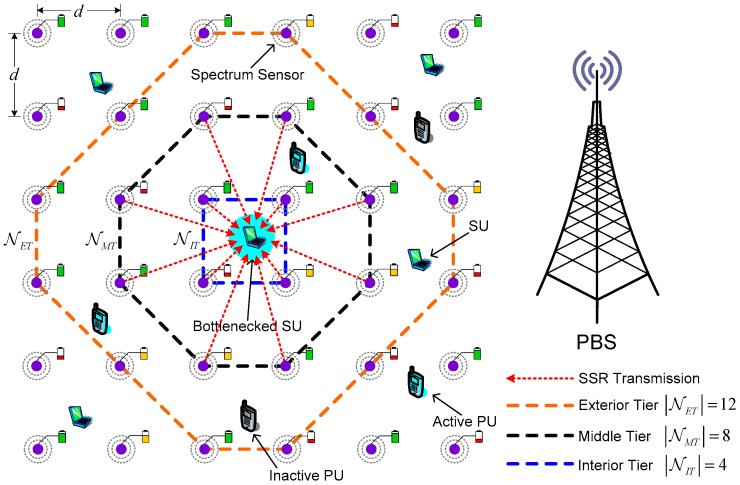
Illustration of the rectangular grid based SN-CRN. SU: secondary user; PU: primary user; PBS: primary base station.

We consider an underlay SN-CRN coexisting with a cellular primary network involving $N_{P}$ PUs and $N_{S}$ SUs in a torus area $\Omega_{S}={\left[0,\sqrt{N_{S}/\rho }\right]}^{2}$ (ρ is the spatial density of SUs) sharing the spectrum within the same frequency band simultaneously, as depicted in Figure 1. Particularly, PUs have the full privilege of accessing their allocated frequency band whereas SUs can opportunistically utilize idle channels unoccupied by the PUs. In the cellular primary network, PUs send their data traffic to the primary base station (PBS) via the licensed uplink channels constituting a channel set $C=\left\{1,2,\cdots ,N_{c}\right\}$. We employ the independent and identically distributed alternating ON-OFF process to model the occupation time length of PUs in uplink channels. Specifically, the OFF state indicates the idle state where the unoccupied uplink channels or the SOPs can be freely occupied by SUs.

In CRANET, SUs denoted by a set $N_{S}=\left\{1,2,\cdots ,N_{S}\right\}$ can only leverage the OFF state to access the SOPs over the idle authorized uplink channels. Due to the randomness of data traffic and the dynamic behavior of PUs, we suppose that the SOPs are available for usage by SU *i* with a probability of $\delta_{i}$, for $i\in N_{S}$. With the aid of the ON-OFF process to characterize the status of the uplink channels, the occupancy probability of the *k*-th uplink channel by PUs can be given by $\alpha_{k}/\left(\alpha_{k}+\beta_{k}\right)$, where $\alpha_{k}$ is probability that the *k*-th uplink channel transits from OFF to ON state, and $\beta_{k}$ is probability that the *k*-th uplink channel transits from ON to OFF state, for k∈C. We also assume that the occupancy probability of uplink channels by PUs can be acquired by SUs through a priori knowledge of the local spectrum sensing. By bearing in mind the mutually independent occupancy probability of the *k*-th uplink channel, the SOP usage probability $\delta_{i}$ of SU *i* during time interval $\left[t_{0},T\right]$ is formulated as follows:

(1) $$\delta_{i}=\prod_{k=1}^{N_{c}}\left(1-\frac{\alpha_{k}}{\alpha_{k}+\beta_{k}}\right),\;i=1,\cdots ,N_{s}$$

Let $\vartheta_{i}\left(t\right)$ and $B_{i}$ denote the amount of data traffic in the buffer of SU *i* at time $t\in \left[t_{0},T\right]$ and the buffer size of SU *i*, respectively. The buffer of SU *i* generally consists of two buffer segments that can hold the offered data load including data traffic injected from upstream SUs and the data sequence of SSR transmitted from SSNs, respectively. Given a time interval Δt, the first buffer segment of SU *i* called as the forward buffer holds the amount of data traffic injected from upstream SUs denoted by $\vartheta_{i}^{F}\left(\Delta t\right)$. Meanwhile, the second buffer segment of SU *i* known as the internal buffer is used to store the amount of the data sequence of SSR transmitted from SSNs denoted by $\vartheta_{i}^{I}\left(\Delta t\right)$. Thus, the amount of aggregate data traffic from upstream SUs and SSNs in the buffer of SU *i* with a time interval Δt can be formulated as $\varphi_{i}\left(\Delta t\right)=\vartheta_{i}^{F}\left(\Delta t\right)+\vartheta_{i}^{I}\left(\Delta t\right)$. Then, for a given time interval Δt, the amount of data traffic $\vartheta_{i}\left(t\right)$ in buffer of SU *i* at time t evolves as follows:

(2) $$\vartheta_{i}\left(t+\Delta t\right)=\min\left\{\vartheta_{i}\left(t\right)+\varphi_{i}\left(\Delta t\right),B_{i}\right\}-\left(\Lambda_{i}\left(\Delta t\right)+\chi_{i}\left(\Delta t\right)\right)$$

where $\Lambda_{i}\left(\Delta t\right)$ and $\chi_{i}\left(\Delta t\right)$ stand for the amount of data traffic successfully delivered by SU *i* and the amount of the data sequence of SSR removed by SU *i* within a time interval Δt, respectively. It is worth noting that the received data sequence of SSR will be removed by SU *i* within a time interval Δt in order to free storage capacity of the internal buffer.

**Remark** **1.** *From Figure 1, there are* $\left|N_{IT}\right|+\left|N_{ET}\right|$ *hop-by-hop fluid flows of the data sequence of SSR injected from SSNs to a single SU (i.e., FC), in addition to the amount of data traffic from upstream SUs. Apparently, this single SU, also known as a possible bottlenecked SU, is a little more inclined to be a congested SU node as a consequence of its buffer overflow. For convenience, the terms bottlenecked SU is used in the following to describe a possible congested SU. It is worth noting that our work in this paper mainly concentrates on how to attain effective congestion mitigation for a single possible congested SU due to buffer overflow caused by the data sequence of SSR injected from SSNs by means of the proposed distributed power control framework for SSNs. However, it is conceivable that the amount of data traffic from upstream SUs may also lead to the congestion of bottlenecked SUs. This problem can be resolved by the specific congestion control technique (e.g., [25]) which is out of the scope of this work.*

### 2.2. Sensor Network Model

As shown in Figure 1, we consider a rectangular grid based sensor network deployed in $\Omega_{S}$ to provide the SSR about real-time spectrum availability information to SUs. Each SSN is equipped with a single omnidirectional antenna, a predefined common control channel (CCC), and an energy detector that continuously senses the entire primary licensed uplink channels through individual local real-time measurement. We suppose that the distributed collaborative spectrum sensing is carried out by multiple collaborating SSNs to enhance the sensing performance. Also, each SU serves as the FC collecting the SSR and then makes a global decision on the availability of the monitored uplink channels via a decision fusion rule, e.g., OR-rule fusion mechanism [7]. All SSNs simultaneously communicate to SUs over a narrowband additive white Gaussian noise (AWGN) multiple-access channel with the channel bandwidth denoted by W. The horizontal or vertical distance between any SSNs in rectangular grid is initialized to be d. According to the location of each SU along with the distance between SSN and the corresponding SU, we define the set of SSNs center around the SU as a tier in rectangular grid. More specifically, the three-tier structure is exploited to organize SSNs into three groups due to the simplicity of implementation as shown in Figure 1, including an interior tier (IT) denoted by a set $N_{IT}=\left\{1,\cdots ,n_{IT}\right\}$ with $n_{IT}=4$, a middle tier (MT) denoted by a set $N_{MT}=\left\{1,\cdots ,n_{MT}\right\}$ with $n_{MT}=8$, and an exterior tier (ET) denoted by a set $N_{ET}=\left\{1,\cdots ,n_{ET}\right\}$ with $n_{ET}=12$.

**Remark** **2.** Because of multiple SUs sharing the authorized uplink channels with PUs, one SSN may also belong to the different tiers based on the presented division criterion to devise the three-tier structure as stated previously. It is important to emphasize that our work in this paper is mainly aimed to study congestion mitigation approaches for one single possible congested SU under the scenario of the single three-tier structure of SSNs. The underlying scenario of the superimposed three-tier structures to organize SSNs is beyond the scope of this work. However, the results about our proposed distributed power control framework for SSNs are easily extendable to the superimposed three-tier structures.

**Figure 2:**
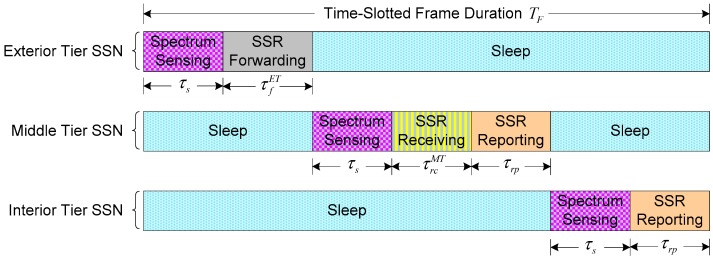
The time-slotted frame structure adopted by dedicated spectrum sensor nodes (SSNs) in time-slot duration $T_{F}$. SSR: spectrum sensing results.

We devise a time-slotted frame structure for sensor network, as illustrated in Figure 2, where each frame with duration $T_{F}$ is divided into different slots according to the types of tiers. Let φ be the number of time-slotted frames within time interval $\left[t_{0},T\right]$. Clearly, the time-slotted frame duration is given by $T_{F}=\left(T-t_{0}\right)/\varphi$. We assume that all SSNs from three tiers perform their operations simultaneously from the beginning of each time-slotted frame. We denote by $\tau_{s}$ the fraction of frame duration $T_{F}$ for the slot of spectrum sensing, and denote by $\tau_{rp}$ the fraction of frame duration $T_{F}$ for the slot of SSR reporting to the bottlenecked SU. We also use $\tau_{f}^{ET}$ to represent the slot of SSR forwarding from ET SSNs to neighbor MT SSNs, and use $\tau_{rc}^{MT}$ to denote the slot of SSR receiving by MT SSNs. During the remaining time, i.e., $T_{F}-\tau_{s}-\tau_{f}^{ET}$ for ET, $T_{F}-\tau_{s}-\tau_{rc}^{MT}-\tau_{rp}$ for MT, and $T_{F}-\tau_{s}-\tau_{rp}$ for IT, SSNs will go into sleep mode to save energy.

During the slot $\tau_{rp}$, the data sequence of the SSR will be transmitted by IT and MT SSNs to bottleneck SU. For analytical simplicity, we assume that the starting time and the terminal time of the slot $\tau_{rp}$ are equal to $t_{0}^{\prime }$ and $t_{0}^{\prime }+\tau_{rp}$ for both IT and MT SSNs, respectively. The instantaneous transmit power of the *m*-th SSN from IT or MT at time $t\in \left[t_{0}^{\prime },t_{0}^{\prime }+\tau_{rp}\right]$, denoted by $p_{m}\left(t\right)$, can be adjusted in a continuous way but is also limited by a maximum value $P_{m}^{\max}$, i.e., $0\leq p_{m}\left(t\right)\leq P_{m}^{\max}$. In the case of the simultaneous communications by IT and MT SSNs over an AWGN multiple-access channel, $p_{m}\left(t\right)$ should satisfy an average power constraint given as follows to mitigate the interference among IT and MT SSNs [26]:

(3) $$\left\{ \begin{array}{ll} \frac{1}{n_{IT}}\sum_{m=1}^{n_{IT}}p_{m}\left(t\right)\leq P_{av}^{IT}, & m\in N_{IT}\\ \frac{1}{n_{MT}}\sum_{m=1}^{n_{MT}}p_{m}\left(t\right)\leq P_{av}^{MT}, & m\in N_{MT} \end{array}\right.$$

where $P_{av}^{IT}$ and $P_{av}^{MT}$ are the total average power assigned to IT and MT SSNs, respectively. We assume that each SSN knows its distance $d_{m\to b}$ from bottleneck SU *b* via the CCC and the channel path gain $h_{m\to b}$ from the *m*-th SSN to the bottlenecked SU *b* can be expressed as $h_{m\to b}={\left(d_{m\to b}\right)}^{-\kappa }$, where κ≥2 is the path-loss exponent and $b\in N_{S}$. Thus, the channel capacity between the *m*-th SSN and bottlenecked SU *b* can be characterized by a concave function of the transmit power and channel conditions as follows [27]:

(4) $$C_{m\to b}=W\log_{2}\left(1+\frac{p_{m}\left(t\right)K{\left|h_{m\to b}\right|}^{2}}{N_{0}W}\right)$$

where K is a constant that depends on the transmission frequency and $N_{0}$ is the noise power spectral density. Under this channel capacity formulation, the signal-to-noise ratio (SNR) between the *m*-th SSN and bottlenecked SU *b* is given by:

(5) $$\gamma_{m}=\frac{p_{m}\left(t\right)K{\left|h_{m\to b}\right|}^{2}}{N_{0}W}$$

**Remark** **3.** *It is assumed that the data sequence of the SSR will be forwarded by ET SSNs to neighbor MT SSNs via a single-hop fashion during the slot* $\tau_{f}^{ET}$*. Let* $t_{0}^{\prime \prime }$ *and* $t_{0}^{\prime \prime }+\tau_{f}^{ET}$ *denote the starting time and the terminal time of the slot* $\tau_{f}^{ET}$*, respectively. The transmit power* $p_{n}\left(t\right)$ *of the n-th SSN from ET at time* $t\in \left[t_{0}^{\prime \prime },t_{0}^{\prime \prime }+\tau_{f}^{ET}\right]$ *is limited to a maximum transmit power* $P_{n}^{\max}$*, for* $0\leq p_{n}\left(t\right)\leq P_{n}^{\max}$ *and* $n\in N_{ET}$*. It is clear that the neighbor MT SSN with the shortest distance will be selected by an ET SSN as the next-hop SSN to save energy during the SSR forwarding period. In other words, the selection metric for the next-hop SSN by an ET SSN is only dependent on the distance between neighbor MT SSN and itself. Owing to the scenario of the rectangular grid based sensor network, it should be admitted that the shortest distance between ET SSN and neighbor MT SSN is equal to* d *for any ET SSN. Therefore, the n-th SSN from ET is inclined to hold the same transmit power* $p_{n}\left(t\right)$ *at time* t*. Under an AWGN multiple-access channel, the average power constraint should also be satisfied for all ET SSNs, i.e.,* $\sum_{n\in N_{ET}}p_{n}\left(t\right)\leq n_{ET}P_{av}^{ET}$*, where* $P_{av}^{ET}$ *is the total average power assigned to ET SSNs. Thus, our work in this paper primarily focuses on the distributed power control for IT and MT SSNs.*

**Remark** **4.** *The channel capacity* $C_{m\to b}$ *in Equation (4) will be rigorously guaranteed if the channel state information (CSI) including the channel path gain* $h_{m\to b}$ *and constant* K *is perfectly known at the m-th SSN which transmits the data sequence of the SSR. In practice, the perfect knowledge of the CSI measured at the m-th SSN side cannot be available because of time-varying wireless channel impairments along with hardware limitations [28,29]. How to model the uncertain relation between the channel path gain* $h_{m\to b}$ *or constant* K *and their estimates by taking into account the effect of imperfect CSI and outage constraint on distributed power control for IT and MT SSNs will be our further work in future.*

### 2.3. Local Spectrum Sensing Model

We denote by ${ℋ}_{1}$ and ${ℋ}_{0}$ the binary hypotheses of the presence and absence of the PU on the uplink channel, respectively. Without loss of generality, we choose the *m*-th SSN from the three-tier structure of sensor network to describe its local spectrum sensing model during the slot $\tau_{s}$. This formulation can be easily extendable to the general case for any SSN from one of the tiers including IT, MT, and ET. The sampled signals that are received at the *m*-th SSN on the *k*-th uplink channel during the slot of spectrum sensing $\tau_{s}$ are given as:

(6) $$y_{m,k}\left(u\right)=\left\{ \begin{array}{ll} h_{m,k}s\left(u\right)+\upsilon_{m,k}\left(u\right), & {ℋ}_{1}\\ \upsilon_{m,k}\left(u\right), & {ℋ}_{0} \end{array}\right.$$

where s(u) denotes the signal from the PU on the *k*-th uplink channel with a sampling frequency $f_{s}$, $\upsilon_{m,k}\left(u\right)$ is the noise at the *m*-th SSN on the *k*-th uplink channel, $h_{m,k}$ is the channel gain between the PU and the *m*-th SSN on the *k*-th uplink channel implying Rayleigh fading. Then the number of samples that is collected by the *m*-th SSN on the *k*-th uplink channel can be defined as $U_{s}=f_{s}\tau_{s}$. We assume that the PU signal s(u) satisfies an independent identically distributed (*i.i.d.*) random process with zero mean and variance $\sigma_{s}^{2}$, and the noise $\upsilon_{m,k}\left(u\right)$ is *i.i.d.* circularly symmetric complex Gaussian with zero mean and variance $\sigma_{\upsilon }^{2}$ [8]. Thus, the received SNR from the PU at the *m*-th SSN on the *k*-th uplink channel is given by $\gamma_{m,k}={\left|h_{m,k}\right|}^{2}\sigma_{s}^{2}/\sigma_{\upsilon }^{2}$. After collecting $U_{s}$ received signal samples on the *k*-th uplink channel, the *m*-th SSN obtains its test statistics given as $Y\left(m,k\right)=\left(1/U_{s}\right)\sum_{u=1}^{U_{s}}{\left|\upsilon_{m,k}\left(u\right)\right|}^{2}$. Let $\varepsilon_{m}$ denote a decision threshold by the *m*-th SSN to decide whether the channel is occupied by the PU. For the *m*-th SSN, the probabilities of detection and false alarm on the *k*-th uplink channel are approximately formulated as follows [8]:

(7) $$p_{d}\left(m,k\right)=\mathrm{Pr}\left(Y\left(m,k\right)>\varepsilon_{m}|{ℋ}_{1}\right)=Q\left(\left(\frac{\varepsilon_{m}}{\sigma_{\upsilon }^{2}}-\gamma_{m,k}-1\right)\sqrt{\frac{U_{s}}{2\gamma_{m,k}+1}}\right)$$

(8) $$p_{f}\left(m,k\right)=\mathrm{Pr}\left(Y\left(m,k\right)>\varepsilon_{m}|{ℋ}_{0}\right)=Q\left(\left(\varepsilon_{m}-1\right)\sqrt{U_{s}}\right)$$

where Q(⋅) denotes the right-tail probability of a normalized Gaussian distribution. Hence, the detection probability set $P_{d}$ and the false alarm probability set $P_{f}$ for the *m*-th SSN over the entire uplink channels can be further expressed as:

(9) $$P_{d}=\left\{p_{d}\left(m,1\right),p_{d}\left(m,2\right),\cdots ,p_{d}\left(m,k\right),\cdots ,p_{d}\left(m,N_{c}\right)\right\}$$

(10) $$P_{f}=\left\{p_{f}\left(m,1\right),p_{f}\left(m,2\right),\cdots ,p_{f}\left(m,k\right),\cdots ,p_{f}\left(m,N_{c}\right)\right\}$$

## 3. Spectrum Sensing Quality Analysis

In this section, our objective is to analyze the spectrum sensing quality of each SSN via a local spectrum sensing model, aiming to provide the quantification result with the emphasis to evaluate the spectrum sensing performance of each SSN. More importantly, the analysis results will be employed to formulate the distributed power control framework for IT and MT SSNs. By revisiting Equations (9) and (10) in local spectrum sensing model, we can observe that the detection probability $p_{d}\left(m,k\right)$ in $P_{d}$ and the false alarm probability $p_{f}\left(m,k\right)$ in $P_{f}$ can be referred to the random variables for the *m*-th SSN under the *k*-th uplink channel due to the uncertainty of the presence and absence of the PU. It is worth noting that the errors in spectrum sensing for a SSN will be generally considered negligible due to imperfect spectrum sensing [27], e.g., misdetection and false alarm caused by hardware capability of SSN and practical time-varying channel conditions. So the errors in spectrum sensing for a SSN will further incur the fact that different uplink channels will hold different probabilities of detection and false alarm. In particular, the higher the detection probability in $P_{d}$, the better the PUs are protected; the lower the false alarm probability in $P_{f}$, the more efficiently the uplink channel can be reutilized by SUs [7]. Based on this observation, the higher the relative divergence between $p_{d}\left(m,k\right)$ and $p_{f}\left(m,k\right)$, the better the performance of local spectrum sensing. It has been revealed that the Kullback–Leibler divergence is an effective measure of how one probability diverges from a second probability [30]. Hence, the relative divergence between $p_{d}\left(m,k\right)$ and $p_{f}\left(m,k\right)$ for the *m*-th SSN under the *k*-th uplink channel can be defined as follows based on a Kullback–Leibler divergence framework:

(11) $$D\left(p_{d}\left(m,k\right)\|p_{f}\left(m,k\right)\right)≜p_{d}\left(m,k\right)\log_{2}\frac{p_{d}\left(m,k\right)}{p_{f}\left(m,k\right)}$$

With respect to the entire set of uplink channels, the relative divergence between $P_{d}$ and $P_{f}$ for the *m*-th SSN can be denoted as:

(12) $$D_{m}(P_{d}\|P_{f})=\sum_{k=1}^{N_{c}}p_{d}\left(m,k\right)\log_{2}\frac{p_{d}\left(m,k\right)}{p_{f}\left(m,k\right)}$$

It is noticeable that the relative divergence between the detection probability and the false alarm probability just reflects the performance of local spectrum sensing by each SSN. Viewed from the SU perspective, we are also interested in the impact of the SOP usage probability on the spectrum sensing quality. To this end, we characterize the spectrum sensing quality factor which can be expressed by a function of two parameters including the relative divergence between $P_{d}$ and $P_{f}$ along with the SOP usage probability $\delta_{b}$ for bottleneck SU *b*. Specifically, the spectrum sensing quality factor ${ℱ}_{m\to b}$ of the *m*-th SSN with respect to bottleneck SU *b* can be defined as:

(13) $$F_{m\to b}≜\delta_{b}\cdot D_{m}\left(P_{d}\|P_{f}\right),\;b\in N_{S}$$

By using mathematical statistics theory, next we start by formulating the detection probability distribution ${ϒ}_{d}\left(m\right)$ and the false alarm probability distribution ${ϒ}_{f}\left(m\right)$, which have been derived from Algorithm 1.

**Algorithm 1:** Generation Procedure of Distribution ${ϒ}_{d}\left(m\right)$ and Distribution ${ϒ}_{f}\left(m\right)$1:**Input**: The detection probability set $P_{d}$ and the false alarm probability set $P_{f}$.2:**Output**: The detection probability distribution ${ϒ}_{d}\left(m\right)=\left\{{ϒ}_{1}^{d}\left(m\right),{ϒ}_{2}^{d}\left(m\right),\cdots ,{ϒ}_{\xi }^{d}\left(m\right)\right\}$
  The false alarm probability distribution ${ϒ}_{f}\left(m\right)=\left\{{ϒ}_{1}^{f}\left(m\right),{ϒ}_{2}^{f}\left(m\right),\cdots ,{ϒ}_{\varsigma }^{f}\left(m\right)\right\}$.
***The detection probability distribution generation***:1:Sort the detection probability $p_{d}\left(m,1\right),p_{d}\left(m,2\right),\cdots ,p_{d}\left(m,N_{c}\right)$ in ascending order and constitute the sorted sequence as ${\hat{p}}_{d}\left(1\right),{\hat{p}}_{d}\left(2\right),\cdots ,{\hat{p}}_{d}\left(N_{c}\right)$.2:$\forall X_{d},Y_{d}>0$, for $X_{d}<{\hat{p}}_{d}\left(1\right)$ and $Y_{d}>{\hat{p}}_{d}\left(N_{c}\right)$.3:Divide interval $\left[X_{d},Y_{d}\right]$ into ξ equal subintervals, i.e., $X_{d}=\mu_{0}^{d}<\mu_{1}^{d}<\mu_{2}^{d}\cdots <\mu_{\xi -1}^{d}<\mu_{\xi }^{d}=Y_{d}$.4:**for**
ℓ=1→ξ
**do**5: $\mu_{\ell }^{d}-\mu_{\ell -1}^{d}=\left(Y_{d}-X_{d}\right)/\xi$.6: Calculate the number of the detection probabilities within subinterval $\left(\mu_{\ell -1}^{d},\mu_{\ell }^{d}\right]$ denoted by $n_{\ell }^{d}$.7: Calculate the probability ${ϒ}_{\ell }^{d}\left(m\right)=n_{\ell }^{d}/N_{c}$.8:**end for**9:**Return:**
${ϒ}_{d}\left(m\right)=\left\{{ϒ}_{1}^{d}\left(m\right),{ϒ}_{2}^{d}\left(m\right),\cdots ,{ϒ}_{\xi }^{d}\left(m\right)\right\}$.
***The false alarm probability distribution generation***:1:Sort the false alarm probability $p_{f}\left(m,1\right),p_{f}\left(m,2\right),\cdots ,p_{f}\left(m,N_{c}\right)$ in ascending order and constitute the sorted sequence as ${\hat{p}}_{f}\left(1\right),{\hat{p}}_{f}\left(2\right),\cdots ,{\hat{p}}_{f}\left(N_{c}\right)$.2:$\forall X_{f},Y_{f}>0$, for $X_{f}<{\hat{p}}_{f}\left(1\right)$ and $Y_{f}>{\hat{p}}_{f}\left(N_{c}\right)$.3:Divide interval $\left[X_{f},Y_{f}\right]$ into ς equal subintervals, *i.e.*, $X_{f}=\mu_{0}^{f}<\mu_{1}^{f}<\mu_{2}^{f}\cdots <\mu_{\varsigma -1}^{f}<\mu_{\varsigma }^{f}=Y_{f}$.4:**for**
ℓ=1→ς
**do**5: $\mu_{\ell }^{f}-\mu_{\ell -1}^{f}=\left(Y_{f}-X_{f}\right)/\varsigma$.6: Calculate the number of the false alarm probabilities within subinterval $\left(\mu_{\ell -1}^{f},\mu_{\ell }^{f}\right]$ denoted by $n_{\ell }^{f}$.7: Calculate the probability ${ϒ}_{\ell }^{f}\left(m\right)=n_{\ell }^{f}/N_{c}$.8:**end for**9:**Return:**
${ϒ}_{f}\left(m\right)=\left\{{ϒ}_{1}^{f}\left(m\right),{ϒ}_{2}^{f}\left(m\right),\cdots ,{ϒ}_{\varsigma }^{f}\left(m\right)\right\}$.

Owing to the fact that the number of the detection probabilities or the false alarm probabilities is calculated under the constraint of $N_{c}$, it is clear that the distributions ${ϒ}_{d}\left(m\right)$ and ${ϒ}_{f}\left(m\right)$ fall into the complete probability distributions, i.e., $\sum_{\ell =1}^{\xi }{ϒ}_{\ell }^{d}\left(m\right)=1$ and $\sum_{\ell =1}^{\varsigma }{ϒ}_{\ell }^{f}\left(m\right)=1$. Apparently, the entropy paradigm should be used for a measure of the uncertainty associated with a random variable of a distribution in information theory [31], and can be also applied to measure the uncertainty of the distributions ${ϒ}_{d}\left(m\right)$ and ${ϒ}_{f}\left(m\right)$. As a result, for the *m*-th SSN over the entire uplink channels, the uncertainty characterization of the distributions ${ϒ}_{d}\left(m\right)$ and ${ϒ}_{f}\left(m\right)$ can be respectively described as based on the entropy modeling framework:

(14) $$H\left({ϒ}_{d}\left(m\right)\right)=-\sum_{\ell =1}^{\xi }{ϒ}_{\ell }^{d}\left(m\right)\log_{2}{ϒ}_{\ell }^{d}\left(m\right)$$

(15) $$H\left({ϒ}_{f}\left(m\right)\right)=-\sum_{\ell =1}^{\varsigma }{ϒ}_{\ell }^{f}\left(m\right)\log_{2}{ϒ}_{\ell }^{f}\left(m\right)$$

In what follows, we are also interested in gaining a better understanding of how to apply this entropy measurement to evaluate the stability of local spectrum sensing. It should be admitted that the entropy tends to be larger when the change of the random variable values in given distribution is disorder or randomness [32]. That is, a more disordered probability distribution will result in larger entropy. Thus, the better performance of spectrum sensing for the *m*-th SSN will bring about the more ordered probability distributions ${ϒ}_{d}\left(m\right)$ and ${ϒ}_{f}\left(m\right)$. In this way, the stability of the distributions ${ϒ}_{d}\left(m\right)$ and ${ϒ}_{f}\left(m\right)$ will decrease because of more disorder for the values of the detection probability and false alarm probability in the distributions ${ϒ}_{d}\left(m\right)$ and ${ϒ}_{f}\left(m\right)$. Based on the insight, we model the stability metric of local spectrum sensing by the relative divergence between the entropy of the detection probability distribution and the entropy of the false alarm probability distribution. Thus, the stability metric of local spectrum sensing for the *m*-th SSN over the entire uplink channels denoted by $H_{m}\left({ϒ}_{d}\left(m\right)\|{ϒ}_{f}\left(m\right)\right)$ can be calculated as follows:

(16) $$H_{m}\left({ϒ}_{d}\left(m\right)\|{ϒ}_{f}\left(m\right)\right)=\left|H\left({ϒ}_{d}\left(m\right)\right)-H\left({ϒ}_{f}\left(m\right)\right)\right|$$

## 4. Distributed Power Control for Spectrum Sensor Nodes

### 4.1. Problem Formulation

It is considered that the channel capacity between the SSNs from IT or MT and a bottlenecked SU is a concave function of the transmit power and channel conditions. Therefore, each SSN is expected to increase the transmit power in the physical layer to provide as much channel capacity that each flow of the data sequence of the SSR requires. However, the higher transmit power will result in the more energy consumption for SSN. Meanwhile, the average power constraint will not be guaranteed for all SSNs if each SSN aims to increase the transmit power. As a consequence, it is necessary to require a tradeoff between channel capacity and energy consumption by achieving an optimal power allocation for all IT and MT SSNs in the physical layer during the slot $\tau_{rp}$. Under the constraint of path-loss of wireless channel, the maximum transmit power of the *m*-th SSN at a distance $d_{m\to b}$ is approximately equal to [33]:

(17) $$P_{m}^{\max}(\mathrm{dBm})=P_{0}(\mathrm{dBm})-10\kappa log_{10}\left(d_{m\to b}/d_{0}\right)$$

where $P_{0}$ is the receiving reference power of bottlenecked SU *b* at a reference distance $d_{0}$. Because of the limitation by a maximum value $P_{m}^{\max}$, the value of power reduction for the *m*-th SSN at time $t\in \left[t_{0}^{\prime },t_{0}^{\prime }+\tau_{rp}\right]$ can be expressed by $P_{m}^{\max}-p_{m}\left(t\right)$. Then the power reduction efficiency for the *m*-th SSN can be written as $\left(P_{m}^{\max}-p_{m}\left(t\right)\right)/P_{m}^{\max}$. To formulate the revenue for power reduction by the *m*-th SSN, we firstly define a pricing factor for power reduction by taking into account both the power reduction efficiency and the stability metric of local spectrum sensing according to the distributions ${ϒ}_{d}\left(m\right)$ and ${ϒ}_{f}\left(m\right)$. More precisely, we define a pricing factor by characterizing an efficiency-to-stability ratio for the *m*-th SSN at time $t\in \left[t_{0}^{\prime },t_{0}^{\prime }+\tau_{rp}\right]$, denoted by ${℘}_{m}^{R}$, to describe the power reduction efficiency under the stability of local spectrum sensing, which can be defined as:

(18) $${℘}_{m}^{R}≜\frac{\left(P_{m}^{\max}-p_{m}\left(t\right)\right)/P_{m}^{\max}}{H_{m}\left({ϒ}_{d}\left(m\right)\|{ϒ}_{f}\left(m\right)\right)}$$

Thus, the revenue of the power reduction for the *m*-th SSN at time $t\in \left[t_{0}^{\prime },t_{0}^{\prime }+\tau_{rp}\right]$ by attaining the product of the pricing factor together with the power reduction value, i.e.,

(19) $$U_{m}^{R}={℘}_{m}^{R}\left(P_{m}^{\max}-p_{m}\left(t\right)\right)$$

From Equations (18) and (19), it is worth remarking that the smaller of stability metric of local spectrum sensing will generate more pricing ${℘}_{m}^{R}$ and also yield more revenue $U_{m}^{R}$ of the power reduction for the *m*-th SSN. On the other hand, it is also a natural idea for the *m*-th SSN to reduce its transmit power to obtain more revenue $U_{m}^{R}$ according to Equation (19). To depict the cost of energy consumption for the *m*-th SSN, we further define another pricing factor for energy consumption, denoted by ${℘}_{m}^{C}$, by considering the spectrum sensing quality factor ${ℱ}_{m\to i}$ as:

(20) $${℘}_{m}^{C}≜e^{-\delta_{i}\cdot D_{m}\left(P_{d}\|P_{f}\right)}$$

Recall that the higher spectrum sensing quality factor will result in the better performance of local spectrum sensing by each SSN. As can be seen from Equation (20), it will be far more realistic to reduce the pricing ${℘}_{m}^{C}$ for energy consumption for the m-th SSN with respect to the better performance of its local spectrum sensing, aiming to balance local spectrum sensing and the energy efficiency of SSN [34]. Let $E_{m}\left(t\right)$ and $E_{A}\left(t\right)$ represent the energy consumption value and the available energy value of the *m*-th SSN at time t, respectively. It is assumed that the available energy of the *m*-th SSN is derived from its battery of limited capacity and the harvesting energy from the renewable energy sources by exploiting the energy harvesting technology. Thus, the energy consumption of the *m*-th SSN evolves according to a linear differential equation given as:

(21) $$\frac{dE_{m}\left(t\right)}{dt}=E_{A}\left(t\right)-\tau_{rp}p_{m}\left(t\right)-E_{m}\left(t\right)$$

Then the cost of energy consumption for the *m*-th SSN at time $t\in \left[t_{0}^{\prime },t_{0}^{\prime }+\tau_{rp}\right]$ can be given as:

(22) $$U_{m}^{C}={℘}_{m}^{C}E_{m}\left(t\right)$$

Therefore, based on the revenue and the cost formulated in Equations (19) and (22), the utility function of the *m*-th SSN at time $t\in \left[t_{0}^{\prime },t_{0}^{\prime }+\tau_{rp}\right]$ can be constructed as follows:

(23) $$U_{m}=U_{m}^{R}-U_{m}^{C}={℘}_{m}^{R}\left(P_{m}^{\max}-p_{m}\left(t\right)\right)-{℘}_{m}^{C}E_{m}\left(t\right)$$

We denote the discount factor by r, for 0<r<1. Our optimization objective is to maximize the utility function $U_{m}$ by choosing optimal transmit power $p_{m}^{OP}\left(t\right)$ of the *m*-th SSN during the slot $\tau_{rp}$ according to ${℘}_{m}^{R}$ and ${℘}_{m}^{C}$, at time $t\in \left[t_{0}^{\prime },t_{0}^{\prime }+\tau_{rp}\right]$, i.e.,

(24) $$\mathrm{Maximize}:\;\int_{t_{0}^{\prime }}^{t_{0}^{\prime }+\tau_{rp}}\left({℘}_{m}^{R}\left(P_{m}^{\max}-p_{m}\left(t\right)\right)-{℘}_{m}^{C}E_{m}\left(t\right)\right)e^{-r\left(t-t_{0}^{\prime }\right)}$$

It is noteworthy that the discount factor r is an exponential factor between 0 and 1 by which the future utility must be multiplied in order to obtain the present value with the underlying structure of differential game theory in mind. Therefore, each SSN is required to maximize its discounted utility $U_{m}$ function by discount factor r, implying that discount factor will have a stronger impact on the utility obtained by each SSN in the future. To this end, the power control problem for all IT and MT SSNs in the physical layer can be formulated as a differential game model defined by $G=\left\{N,{\left\{p_{m}^{OP}\left(t\right)\right\}}_{m\in N},E_{m}^{OP}\left(t\right),{\left\{U_{m}\right\}}_{m\in N}\right\}$, where $N=N_{IT}\cup N_{MT}$ is the set of players involving all IT and MT SSNs, $p_{m}^{OP}\left(t\right)$ is the strategy of player *m*, ${\left\{p_{m}^{OP}\left(t\right)\right\}}_{m\in N}$ is the set of strategies or strategy space related to all players, $E_{m}^{OP}\left(t\right)$ is the state variable associated with optimal transmit power $p_{m}^{OP}\left(t\right)$, and ${\left\{U_{m}\right\}}_{m\in N}$ is the set of utility function of all players with their strategies.

### 4.2. Noncooperative Optimal Solution

We formulate a dynamic optimization problem P1 to derive the optimal solution to the differential game model G with the objective of the utility function maximization problem under the linear differential equation constraint of the energy consumption for the *m*-th SSN. From Equations (21) and (24), the problem P1 can be formulated as:

(25) $$\begin{array}{ccc} P1: & \underset{p_{m}\left(t\right),m\in N}{\max} & \int_{t_{0}^{\prime }}^{t_{0}^{\prime }+\tau_{rp}}\left({℘}_{m}^{R}\left(P_{m}^{\max}-p_{m}\left(t\right)\right)-{℘}_{m}^{C}E_{m}\left(t\right)\right)e^{-r\left(t-t_{0}^{\prime }\right)}dt\\ & s.t. & \frac{dE_{m}\left(t\right)}{dt}=E_{A}\left(t\right)-\tau_{rp}p_{m}\left(t\right)-E_{m}\left(t\right) \end{array}$$

For the noncooperation scenario if all the players play noncooperatively, we aim at deriving an optimal solution to the problem P1 in the distributed noncooperative power control (NoCoPC) problem for all IT and MT SSNs by employing the theory of dynamic programming developed by Bellman. Note that the players can abandon the cooperation due to their selfishness and own interests in the NoCoPC problem, e.g., the selfish behavior in forwarding the data sequence of the SSR to bottleneck SU. Specifically, we employ $p_{m}^{NC}\left(t\right)$ to represent the noncooperative optimal solution to the problem P1, and assume that there exists a continuously differentiable function $V_{m}^{NC}\left(p_{m},E_{m}\right)$ satisfying the following partial differential equation:

(26) $$rV_{m}^{NC}\left(p_{m},E_{m}\right)=\max\left({℘}_{m}^{R}\left(P_{m}^{\max}-p_{m}\left(t\right)\right)-{℘}_{m}^{C}E_{m}\left(t\right)+\frac{\partial V_{m}^{NC}\left(p_{m},E_{m}\right)}{\partial E_{m}}\left(E_{A}\left(t\right)-\tau_{rp}p_{m}\left(t\right)-E_{m}\left(t\right)\right)\right)$$

**Proposition** **1.** *The function* $V_{m}^{NC}\left(p_{m},E_{m}\right)$ *for the m-th SSN in Equation (26) should be subject to the partial differential equation constraint as follows:*

(27) $$\frac{\partial V_{m}^{NC}\left(p_{m},E_{m}\right)}{\partial E_{m}}=-\frac{{℘}_{m}^{C}}{r+1}$$

**Proof.**  See Appendix A. ☐

**Proposition** **2.** *The noncooperative optimal solution* $p_{m}^{NC}\left(t\right)$ *constitutes a Nash equilibrium solution to the problem* P1 *if and only if the optimal transmit power for the m-th SSN can be expressed as:*

(28) $$p_{m}^{NC}\left(t\right)=P_{m}^{\max}\left(1-\frac{H_{m}\left({ϒ}_{d}\left(m\right)\|{ϒ}_{f}\left(m\right)\right)\tau_{rp}}{2\left(r+1\right)e^{\delta_{i}\cdot D_{m}\left(P_{d}\|P_{f}\right)}}\right)$$

**Proof.**  Recall the following expression of the noncooperative optimal solution in Equation (A1) on the basis of Proposition 1. By substituting the expression of function $V_{m}^{NC}\left(p_{m},E_{m}\right)$ in Equation (27) into Equation (A1), we can easily obtain the noncooperative optimal transmit power $p_{m}^{NC}\left(t\right)$ which constitutes a Nash equilibrium solution to the problem P1 can be formulated by Equation (28). ☐

By observing Proposition 2, it is clear that an increased discount factor r will enhance the noncooperative optimal transmit power $p_{m}^{NC}\left(t\right)$ for the *m*-th SSN. From Equation (28), the noncooperative optimal transmit power vector $P^{NC}$ for all IT and MT SSNs in the NoCoPC problem can be further combined as:

(29) $$P^{NC}=\left\{\underset{\mathrm{for}\;n_{IT}\;\mathrm{IT}\;\mathrm{SSNs}}{\underset{︸}{p_{1}^{NC}\left(t\right),\cdots ,p_{\left|N_{IT}\right|}^{NC}\left(t\right)}},\overset{\mathrm{for}\;n_{MT}\;\mathrm{MT}\;\mathrm{SSNs}}{\overset{︷}{p_{\left|N_{IT}\right|+1}^{NC}\left(t\right),\cdots ,p_{\left|N_{IT}\right|+\left|N_{MT}\right|}^{NC}\left(t\right)}}\right\}$$

Given the noncooperative optimal solution $p_{m}^{NC}\left(t\right)$, by substituting $V_{m}^{NC}\left(p_{m},E_{m}\right)$ and $p_{m}^{NC}\left(t\right)$ into Equation (26), the function $V_{m}^{NC}\left(p_{m}^{NC}\left(t\right),E_{m}^{NC}\left(t\right)\right)$ for the *m*-th SSN is further given by:

(30) $$V_{m}^{NC}\left(p_{m}^{NC}\left(t\right),E_{m}^{NC}\left(t\right)\right)=\frac{{℘}_{m}^{C}}{r\left(r+1\right)}\left(\tau_{rp}P_{m}^{\max}-\frac{P_{m}^{\max}H_{m}\left({ϒ}_{d}\left(m\right)\|{ϒ}_{f}\left(m\right)\right)\tau_{rp}^{2}{℘}_{m}^{C}}{4\left(r+1\right)}-E_{A}\left(t\right)-rE_{m}^{NC}\left(t\right)\right)$$

where $E_{m}^{NC}\left(t\right)$ is the noncooperative optimal energy consumption value of the *m*-th SSN at time t.

### 4.3. Cooperative Optimal Solution

In this subsection, we move on to explore the distributed cooperative power control (CoPC) problem for all IT and MT SSNs by building up the cooperation scenario if all the players play cooperatively via the differential game model G. Note that this is a natural idea for the players aiming to achieve an optimal power allocation through full cooperation for their common interests. Under this scenario, our optimization objective is to maximize the sum of the utility functions of all players (i.e., $\sum_{m\in N}U_{m}$) while the linear differential equation constraint of the energy consumption should also be satisfied for the *m*-th SSN. To be specific, we formulate a dynamic optimization problem P2 as follows to attain the objective of maximizing the sum of the utility functions of all players:

(31) $$\begin{array}{ccc} P2: & \underset{p_{1}\left(t\right),\cdots ,p_{m}\left(t\right),\cdots ,p_{\left|N\right|}\left(t\right),m\in N}{\max} & \sum_{m\in N}\int_{t_{0}^{\prime }}^{t_{0}^{\prime }+\tau_{rp}}\left({℘}_{m}^{R}\left(P_{m}^{\max}-p_{m}\left(t\right)\right)-{℘}_{m}^{C}E_{m}\left(t\right)\right)e^{-r\left(t-t_{0}^{\prime }\right)}dt\\ & s.t. & \frac{dE_{m}\left(t\right)}{dt}=E_{A}\left(t\right)-\tau_{rp}p_{m}\left(t\right)-E_{m}\left(t\right) \end{array}$$

Under the cooperation scenario, we use $p_{m}^{C}\left(t\right)$ to represent the cooperative optimal solution to the problem P2, and assume that there exists a continuously differentiable function $W_{m}^{C}\left(p_{m},E_{m}\right)$ satisfying the following partial differential equation:

(32) $$rW_{m}^{C}\left(p_{m},E_{m}\right)=\max\left(\sum_{m\in N}\left({℘}_{m}^{R}\left(P_{m}^{\max}-p_{m}\left(t\right)\right)-{℘}_{m}^{C}E_{m}\left(t\right)\right)+\frac{\partial W_{m}^{C}\left(p_{m},E_{m}\right)}{\partial E_{m}}\left(E_{A}\left(t\right)-\tau_{rp}p_{m}\left(t\right)-E_{m}\left(t\right)\right)\right)$$

**Proposition** **3.** *The function* $W_{m}^{C}\left(p_{m},E_{m}\right)$ *for the m-th SSN in Equation (32) should be subject to the partial differential equation constraint as follows:*

(33) $$\frac{\partial W_{m}^{C}\left(p_{m},E_{m}\right)}{\partial E_{m}}=-\frac{\sum_{m\in N}{℘}_{m}^{C}}{r+1}$$

**Proof.**  See Appendix B. ☐

**Proposition** **4.** *The cooperative optimal solution* $p_{m}^{C}\left(t\right)$ *to the problem* P2 *if and only if the optimal transmit power for the m-th SSN can be expressed as*:

(34) $$p_{m}^{C}\left(t\right)=P_{m}^{\max}\left(1-\frac{H_{m}\left({ϒ}_{d}\left(m\right)\|{ϒ}_{f}\left(m\right)\right)\tau_{rp}}{2\left(r+1\right)\sum_{m\in N}e^{\delta_{i}\cdot D_{m}\left(P_{d}\|P_{f}\right)}}\right)$$

**Proof.**  Substituting the expression of function $W_{m}^{C}\left(p_{m},E_{m}\right)$ in Equation (33) into Equation (A4), and applying the result of the indicated maximization operation in Equation (A4), we derive the cooperative optimal solution $p_{m}^{C}\left(t\right)$ to the problem P2 as in Equation (34). ☐

Similar to Proposition 2, it is also revealed that an increased discount factor r will enhance the cooperative optimal transmit power $p_{m}^{C}\left(t\right)$ for the *m*-th SSN. According to Equation (34), the cooperative optimal transmit power vector $P^{C}$ for all IT and MT SSNs in the CoPC problem can be also combined as follows:

(35) $$P^{C}=\left\{\underset{\mathrm{for}\;n_{IT}\;\mathrm{IT}\;\mathrm{SSNs}}{\underset{︸}{p_{1}^{C}\left(t\right),\cdots ,p_{\left|N_{IT}\right|}^{C}\left(t\right)}},\overset{\mathrm{for}\;n_{MT}\;\mathrm{MT}\;\mathrm{SSNs}}{\overset{︷}{p_{\left|N_{IT}\right|+1}^{C}\left(t\right),\cdots ,p_{\left|N_{IT}\right|+\left|N_{MT}\right|}^{C}\left(t\right)}}\right\}$$

Define the notation $\Psi_{m}=P_{m}^{\max}H_{m}\left({ϒ}_{d}\left(m\right)\|{ϒ}_{f}\left(m\right)\right)$ for simplicity in the following. Given the cooperative optimal solution $p_{m}^{C}\left(t\right)$, by plugging $W_{m}^{C}\left(p_{m},E_{m}\right)$ and $p_{m}^{C}\left(t\right)$ into Equation (32), the function $W_{m}^{C}\left(p_{m}^{C}\left(t\right),E_{m}^{C}\left(t\right)\right)$ for the *m*-th SSN is further given by:

(36) $$\begin{array}{ll} W_{m}^{C}\left(p_{m}^{C}\left(t\right),E_{m}^{C}\left(t\right)\right) & =\frac{\tau_{rp}^{2}}{4r{\left(r+1\right)}^{2}}\left(\sum_{m\in N}\left(\Psi_{m}{\left(\sum_{m\in N}{℘}_{m}^{C}\right)}^{2}\right)-2\Psi_{m}{\left(\sum_{m\in N}{℘}_{m}^{C}\right)}^{2}\right)\\ & +\frac{\left(E_{m}^{C}\left(t\right)-E_{A}\left(t\right)\right)\sum_{m\in N}{℘}_{m}^{C}}{r\left(r+1\right)}+\frac{\tau_{rp}P_{m}^{\max}\sum_{m\in N}{℘}_{m}^{C}}{r}-\frac{1}{r}\sum_{m\in N}\left({℘}_{m}^{C}E_{m}^{C}\left(t\right)\right) \end{array}$$

where $E_{m}^{C}\left(t\right)$ is the cooperative optimal energy consumption value of the *m*-th SSN at time t.

By using $P^{NC}$ of the NoCoPC problem and $P^{C}$ of the CoPC problem, we then design a distributed optimal transmit power adjustment (OTPA) algorithm as stated in Algorithm 2. Recall that the pricing factor ${℘}_{m}^{R}$ is employed to characterize the efficiency-to-stability ratio for the *m*-th SSN, for m∈N, aiming to describe the power reduction efficiency under the stability of local spectrum sensing. Thus, in order to ensure the convergence of optimal transmit power adjustment, we design the average efficiency-to-stability ratio ${\bar{\varpi }}_{m}$ as a scaling coefficient in Algorithm 2 as follows:

(37) $${\bar{\varpi }}_{m}=\left\{ \begin{array}{ll} \frac{{℘}_{m}^{R}}{\sum_{m=1}^{n_{IT}}{℘}_{m}^{R}}, & m\in N_{IT}\\ \frac{{℘}_{m}^{R}}{\sum_{m=n_{IT}+1}^{n_{IT}+n_{MT}}{℘}_{m}^{R}}, & m\in N_{MT} \end{array}\right.$$

It is worth remarking that the adjusted optimal transmit power will be updated with respect ro the scaling coefficient ${\bar{\varpi }}_{m}$. In particular, the adjusted optimal transmit power for all IT and MT SSNs via OTPA algorithm can be made locally while guaranteeing that the condition of the average power constraint as given in Equation (3) is satisfied.

**Algorithm 2**: Distributed Optimal Transmit Power Adjust Algorithm1:**Input**: $P^{NC}$ and $P^{C}$.2:**Output**: $\left\{\underset{1\to n_{IT}}{\underset{︸}{{\tilde{p}}_{1}^{NC}\left(t\right),\cdots ,{\tilde{p}}_{n_{IT}}^{NC}\left(t\right)}},\overset{n_{IT}+1\to n_{IT}+n_{MT}}{\overset{︷}{{\tilde{p}}_{n_{IT}+1}^{NC}\left(t\right),\cdots ,{\tilde{p}}_{n_{IT}+n_{MT}}^{NC}\left(t\right)}}\right\}$ and $\left\{\underset{1\to n_{IT}}{\underset{︸}{{\tilde{p}}_{1}^{C}\left(t\right),\cdots ,{\tilde{p}}_{n_{IT}}^{C}\left(t\right)}},\overset{n_{IT}+1\to n_{IT}+n_{MT}}{\overset{︷}{{\tilde{p}}_{n_{IT}+1}^{C}\left(t\right),\cdots ,{\tilde{p}}_{n_{IT}+n_{MT}}^{C}\left(t\right)}}\right\}$.3:**Initialization**: $\left\{P_{1}^{\max},\cdots ,P_{m}^{\max},\cdots ,P_{\left|N\right|}^{\max}\right\}$, $\left\{{℘}_{1}^{R},\cdots ,{℘}_{m}^{R},\cdots ,{℘}_{\left|N\right|}^{R}\right\}$, $\left\{{℘}_{1}^{C},\cdots ,{℘}_{m}^{C},\cdots ,{℘}_{\left|N\right|}^{C}\right\}$.4:**for**
m=1→|N|
**do**5: ${\tilde{p}}_{m}^{NC}\left(t\right)\leftarrow p_{m}^{NC}\left(t\right)$ using Equation (28), and ${\tilde{p}}_{m}^{C}\left(t\right)\leftarrow p_{m}^{C}\left(t\right)$ using Equation (34).6:**end for**7:**if**
$\sum_{m=1}^{n_{IT}}{\tilde{p}}_{m}^{NC}\left(t\right)>n_{IT}P_{av}^{IT}$ or $\sum_{m=1}^{n_{IT}}{\tilde{p}}_{m}^{C}\left(t\right)>n_{IT}P_{av}^{IT}$
**then**8: **repeat**9:  **for**
$m=1\to n_{IT}$
**do**10:   ${\tilde{p}}_{m}^{NC}\left(t\right)\leftarrow \left({℘}_{m}^{R}/\sum_{m=1}^{n_{IT}}{℘}_{m}^{R}\right)\times {\tilde{p}}_{m}^{NC}\left(t\right)$ when $\sum_{m=1}^{n_{IT}}{\tilde{p}}_{m}^{NC}\left(t\right)>n_{IT}P_{av}^{IT}$.11:   ${\tilde{p}}_{m}^{C}\left(t\right)\leftarrow \left({℘}_{m}^{R}/\sum_{m=1}^{n_{IT}}{℘}_{m}^{R}\right)\times {\tilde{p}}_{m}^{C}\left(t\right)$ when $\sum_{m=1}^{n_{IT}}{\tilde{p}}_{m}^{C}\left(t\right)>n_{IT}P_{av}^{IT}$.12:  **end**13: **until**
$\sum_{m=1}^{n_{IT}}{\tilde{p}}_{m}^{NC}\left(t\right)>n_{IT}P_{av}^{IT}$ and $\sum_{m=1}^{n_{IT}}{\tilde{p}}_{m}^{C}\left(t\right)>n_{IT}P_{av}^{IT}$14:**end if**15:**if**
$\sum_{m=n_{IT}+1}^{n_{IT}+n_{MT}}{\tilde{p}}_{m}^{NC}\left(t\right)>n_{MT}P_{av}^{MT}$ or $\sum_{m=n_{IT}+1}^{n_{IT}+n_{MT}}{\tilde{p}}_{m}^{C}\left(t\right)>n_{MT}P_{av}^{MT}$
**then**16: **repeat**17:  **for**
$m=n_{IT}+1\to n_{IT}+n_{MT}$
**do**18:   ${\tilde{p}}_{m}^{NC}\left(t\right)\leftarrow \left({℘}_{m}^{R}/\sum_{m=n_{IT}+1}^{n_{IT}+n_{MT}}{℘}_{m}^{R}\right)\times {\tilde{p}}_{m}^{NC}\left(t\right)$ when $\sum_{m=n_{IT}+1}^{n_{IT}+n_{MT}}{\tilde{p}}_{m}^{NC}\left(t\right)>n_{MT}P_{av}^{MT}$.19:   ${\tilde{p}}_{m}^{C}\left(t\right)\leftarrow \left({℘}_{m}^{R}/\sum_{m=n_{IT}+1}^{n_{IT}+n_{MT}}{℘}_{m}^{R}\right)\times {\tilde{p}}_{m}^{C}\left(t\right)$ when $\sum_{m=n_{IT}+1}^{n_{IT}+n_{MT}}{\tilde{p}}_{m}^{C}\left(t\right)>n_{MT}P_{av}^{MT}$.20:  **end**21: **until**
$\sum_{m=n_{IT}+1}^{n_{IT}+n_{MT}}{\tilde{p}}_{m}^{NC}\left(t\right)>n_{MT}P_{av}^{MT}$ and $\sum_{m=n_{IT}+1}^{n_{IT}+n_{MT}}{\tilde{p}}_{m}^{C}\left(t\right)>n_{MT}P_{av}^{MT}$22:**end if**

## 5. Congestion Mitigation for Bottlenecked Secondary User

**Figure 3:**
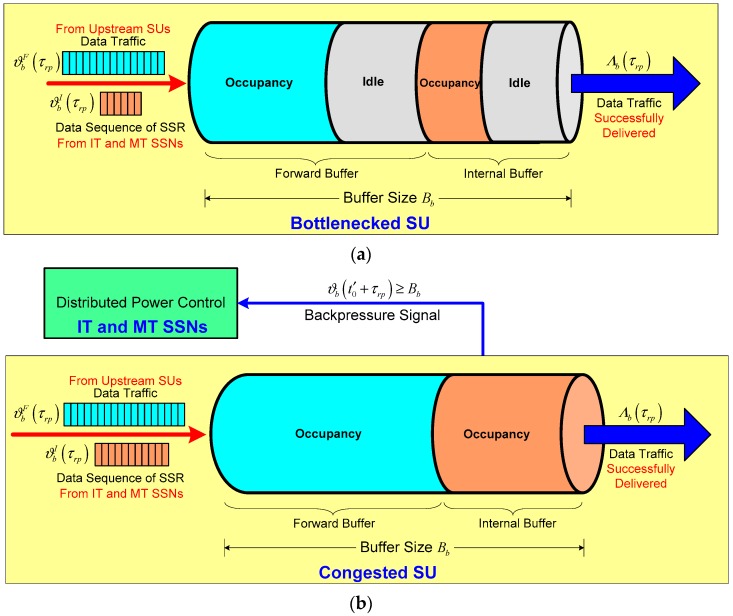
Illustration of buffer based congestion detection at bottlenecked secondary user (SU) *b*. (**a**) Normal status without buffer overflow; (**b**) Congestion occurrence due to buffer overflow.

We have shown that the bottlenecked SU is a little more inclined to be a congested SU node during the slot $\tau_{rp}$ due to its buffer overflow under the scenario of the hop-by-hop fluid flows of the data sequence of the SSR injected from $\left|N_{IT}\right|+\left|N_{ET}\right|$ IT and MT SSNs to this bottlenecked SU. We use the buffer size $B_{b}$ to denote the buffer saturation value of bottlenecked SU *b*. Recall that the amount of data traffic $\vartheta_{b}\left(t_{0}^{\prime }\right)$ in buffer of SU *b* at time $t_{0}^{\prime }$ evolves according to Equation (2) for the given slot $\tau_{rp}$. As depicted in Figure 3a, the buffer of bottlenecked SU *b* is composed of two buffer segments, i.e., the forward buffer that holds data traffic injected from upstream SUs denoted by $\vartheta_{b}^{F}\left(\tau_{rp}\right)$, and the internal buffer that stores the data sequence of SSR transmitted from IT and MT SSNs denoted by $\vartheta_{b}^{I}\left(\tau_{rp}\right)$. Normally, there exist idle segments within the forward buffer or the internal buffer of bottlenecked SU *b* when $\vartheta_{b}\left(t_{0}^{\prime }+\tau_{rp}\right)<B_{b}$. This phenomenon is referred to the normal status where the offered data load does not exceed available buffer capacity of bottleneck SU *b* because of its higher data rate to deliver the amount of data traffic to other SUs and remove the amount of the data sequence of SSR during the slot $\tau_{rp}$.

However, congestion in bottlenecked SU *b* will occur when the offered data load exceeds its available buffer capacity due to buffer overflow imposed by the data load involving the data sequence of the SSR injected from IT and MT SSNs together with the data traffic from upstream SUs. We assume that the congestion detection information of bottlenecked SU *b* can be transferred back to the *m*-th SSN by means of a hop-by-hop backpressure signal via the CCC form bottlenecked SU *b* to the *m*-th SSN. As a result, bottlenecked SU *b* is said to be a congested SU if and only if the amount of its current data traffic $\vartheta_{b}\left(t_{0}^{\prime }+\tau_{rp}\right)\geq B_{b}$ during the slot $\tau_{rp}$, as shown in Figure 3b. We further assume that the *m*-th SSN has the data sequence of the SSR with $\Xi_{m}$ bits to transmit at time $t\in \left[t_{0}^{\prime },t_{0}^{\prime }+\tau_{rp}\right]$. According to [24], the data sequence of SSR with $\Xi_{m}$ bits to transmit from the *m*-th SSN to bottlenecked SU *b* during the slot $\tau_{rp}$ under the transmit power $p_{m}\left(t\right)$ can be approximately expressed as follows based on channel capacity $C_{m\to b}$ given by Equation (4):

(38) $$\Xi_{m}=\tau_{rp}W\log_{2}\left(1+\frac{p_{m}\left(t\right)K{\left|h_{m\to b}\right|}^{2}}{N_{0}W}\right)$$

Clearly, $\Xi_{m}$ depends on channel capacity $C_{m\to b}$ and is also a function of the transmit power $p_{m}\left(t\right)$. For ease of exposition, we use $\Xi_{m}\left(p_{m}\left(t\right)\right)$ to denote the amount of bits of the data sequence of SSR transmitted from the *m*-th SSN to bottlenecked SU *b* under transmit power $p_{m}\left(t\right)$. Therefore, the aggregated amount of bits of the data sequence of SSR from all $\left|N_{IT}\right|+\left|N_{ET}\right|$ IT and MT SSNs to bottleneck SU *b* during the slot $\tau_{rp}$ under transmit power $p_{m}\left(t\right)$ is given as $\sum_{m\in N}\Xi_{m}\left(p_{m}\left(t\right)\right)$, for $N=N_{IT}\cup N_{MT}$. Recall that the internal buffer of bottlenecked SU *b* is used to store the amount of the data sequence of SSR transmitted from SSNs denoted by $\vartheta_{b}^{I}\left(\tau_{rp}\right)$ during the slot $\tau_{rp}$ by using Equation (2). Hence, within the time interval $\tau_{rp}$, $\vartheta_{b}^{I}\left(\tau_{rp}\right)$ of bottlenecked SU *b* can be further formulated by:

(39) $$\vartheta_{b}^{I}\left(\tau_{rp}\right)=\sum_{m\in N}\Xi_{m}\left(p_{m}\left(t\right)\right)-\chi_{b}\left(\tau_{rp}\right)$$

where $\chi_{b}\left(\tau_{rp}\right)$ is the amount of the data sequence of SSR removed by bottlenecked SU *b* within a time interval $\tau_{rp}$.

**Figure 4:**
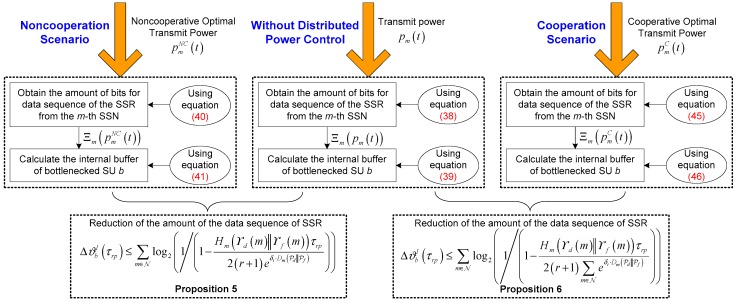
The block diagram of the key point to achieve congestion mitigation for bottleneck secondary user.

So far, we have mathematically derived the amount of the data sequence of SSR transmitted from SSNs to bottlenecked SU *b* under the condition that the *m*-th SSN employs the transmit power $p_{m}\left(t\right)$, for m∈N. Next, we are concerned with how to mitigate the congestion of bottlenecked SU *b* by the aid of the proposed distributed power control framework for IT and MT SSNs. The basic idea of congestion mitigation for bottlenecked SU *b* is to alleviate its buffer load because of the accumulated amount of the data sequence of the SSR transmitted from IT and MT SSNs. It is uncovered that the transmit power of the *m*-th SSN may have a bearing on $\Xi_{m}$ from Equation (38). Our objective is to leverage the distributed power control to reduce the amount of bits of the data sequence of the SSR transmitted from the *m*-th SSN to bottlenecked SU *b*, in order to lower the amount of the data sequence of the SSR in the internal buffer of bottlenecked SU *b*. This operation will further ensure that offered data load does not exceed available buffer capacity of bottlenecked SU *b*, i.e., $\vartheta_{b}\left(t_{0}^{\prime }+\tau_{rp}\right)<B_{b}$. The key point to achieve congestion mitigation for bottlenecked SU *b* is established with a block diagram shown in Figure 4. It is worth remarking that the amount of the data sequence of SSR in the internal buffer of bottlenecked SU *b* can be effectively reduced by the proposed distributed power control framework for IT and MT SSNs under the noncooperation and cooperation scenarios. Conceptually, the reduction of the amount of the data sequence of SSR will release the capacity of the internal buffer for bottlenecked SU *b*, which naturally further attains the congestion mitigation for bottlenecked SU *b*. In the following, we analyze the impact of optimal transmit power for the *m*-th SSN on the reduction of the amount of the data sequence of SSR in the internal buffer of bottleneck SU *b* rigorously from the noncooperation and cooperation cases.

(1) *Noncooperative Optimal Transmit Power Case*: In this case, suppose, without loss of generality, that $\tau_{rp}W≜1$. By replacing $p_{m}\left(t\right)$ in Equation (38) with $p_{m}^{NC}\left(t\right)$ in Equation (28), $\Xi_{m}\left(p_{m}^{NC}\left(t\right)\right)$ for the *m*-th SSN within a time interval $\tau_{rp}$ can be expressed as:

(40) $$\begin{array}{ll} \Xi_{m}\left(p_{m}^{NC}\left(t\right)\right) & =\tau_{rp}W\log_{2}\left(1+\frac{p_{m}^{NC}\left(t\right)K{\left|h_{m\to b}\right|}^{2}}{N_{0}W}\right)\\ & \approx \log_{2}\left(\frac{p_{m}^{NC}\left(t\right)K{\left|h_{m\to b}\right|}^{2}}{N_{0}W}\right)\\ & \approx \log_{2}\left(P_{m}^{\max}\left(1-\frac{H_{m}\left({ϒ}_{d}\left(m\right)\|{ϒ}_{f}\left(m\right)\right)\tau_{rp}}{2\left(r+1\right)e^{\delta_{i}\cdot D_{m}\left(P_{d}\|P_{f}\right)}}\right)\frac{K{\left|h_{m\to b}\right|}^{2}}{N_{0}W}\right) \end{array}$$

By plugging $\Xi_{m}\left(p_{m}^{NC}\left(t\right)\right)$ into Equation (39), we have:

(41) $$\vartheta_{b}^{I}\left(\tau_{rp}\right)=\underset{\mathrm{for}\;n_{IT}\;\mathrm{IT}\;\mathrm{SSNs}}{\underset{︸}{\sum_{m=1}^{n_{IT}}\Xi_{m}\left(p_{m}^{NC}\left(t\right)\right)}}+\underset{\mathrm{for}\;n_{MT}\;\mathrm{MT}\;\mathrm{SSNs}}{\underset{︸}{\sum_{m=n_{IT}+1}^{n_{IT}+n_{MT}}\Xi_{m}\left(p_{m}^{NC}\left(t\right)\right)}}-\chi_{b}\left(\tau_{rp}\right)$$

Thus, the reduction of the amount of the data sequence of SSR in the internal buffer of bottlenecked SU *b*, denoted by $\Delta \vartheta_{b}^{I}\left(\tau_{rp}\right)$, is equivalent to:

(42) $$\begin{array}{ll} \Delta \vartheta_{b}^{I}\left(\tau_{rp}\right) & =\sum_{m=1}^{n_{IT}}\left(\Xi_{m}\left(p_{m}\left(t\right)\right)-\Xi_{m}\left(p_{m}^{NC}\left(t\right)\right)\right)+\sum_{m=n_{IT}+1}^{n_{IT}+n_{MT}}\left(\Xi_{m}\left(p_{m}\left(t\right)\right)-\Xi_{m}\left(p_{m}^{NC}\left(t\right)\right)\right)\\ & \approx \sum_{m\in N}\log_{2}\left(\frac{p_{m}\left(t\right)}{p_{m}^{NC}\left(t\right)}\right) \end{array}$$

**Proposition** **5.** *Under the condition of the transmit power* $p_{m}\left(t\right)=P_{m}^{\max}$ *for the m-th SSN,* $\Delta \vartheta_{b}^{I}\left(\tau_{rp}\right)$ *satisfies the following upper bound:*

(43) $$\Delta \vartheta_{b}^{I}\left(\tau_{rp}\right)\leq \sum_{m\in N}\log_{2}\left(1/\left(1-\frac{H_{m}\left({ϒ}_{d}\left(m\right)\|{ϒ}_{f}\left(m\right)\right)\tau_{rp}}{2\left(r+1\right)e^{\delta_{i}\cdot D_{m}\left(P_{d}\|P_{f}\right)}}\right)\right)$$

**Proof.**  Since $p_{m}\left(t\right)=P_{m}^{\max}$. Under this condition, using Equation (28), we have:

(44) $$\begin{array}{ll} \Delta \vartheta_{b}^{I}\left(\tau_{rp}\right) & \approx \sum_{m\in N}\log_{2}\left(\frac{p_{m}\left(t\right)}{p_{m}^{NC}\left(t\right)}\right)\\ & \leq \sum_{m\in N}\log_{2}\left(\frac{P_{m}^{\max}}{p_{m}^{NC}\left(t\right)}\right)\\ & =\sum_{m\in N}\log_{2}\left(1/\left(1-\frac{H_{m}\left({ϒ}_{d}\left(m\right)\|{ϒ}_{f}\left(m\right)\right)\tau_{rp}}{2\left(r+1\right)e^{\delta_{i}\cdot D_{m}\left(P_{d}\|P_{f}\right)}}\right)\right) \end{array}$$

Then we obtain the upper bound in Equation (43), thus completing the proof. ☐

(2) *Cooperative Optimal Transmit Power Case*: In this case, with the assumption $\tau_{rp}W≜1$, by replacing $p_{m}\left(t\right)$ in Equation (38) with $p_{m}^{C}\left(t\right)$ in Equation (34), $\Xi_{m}\left(p_{m}^{C}\left(t\right)\right)$ for the *m*-th SSN within a time interval $\tau_{rp}$ can be given by:

(45) $$\begin{array}{ll} \Xi_{m}\left(p_{m}^{C}\left(t\right)\right) & =\tau_{rp}W\log_{2}\left(1+\frac{p_{m}^{C}\left(t\right)K{\left|h_{m\to b}\right|}^{2}}{N_{0}W}\right)\\ & \approx \log_{2}\left(\frac{p_{m}^{C}\left(t\right)K{\left|h_{m\to b}\right|}^{2}}{N_{0}W}\right)\\ & \approx \log_{2}\left(P_{m}^{\max}\left(1-\frac{H_{m}\left({ϒ}_{d}\left(m\right)\|{ϒ}_{f}\left(m\right)\right)\tau_{rp}}{2\left(r+1\right)\sum_{m\in N}e^{\delta_{i}\cdot D_{m}\left(P_{d}\|P_{f}\right)}}\right)\frac{K{\left|h_{m\to b}\right|}^{2}}{N_{0}W}\right) \end{array}$$

By substituting $\Xi_{m}\left(p_{m}^{C}\left(t\right)\right)$ into Equation (39), we obtain:

(46) $$\vartheta_{b}^{I}\left(\tau_{rp}\right)=\underset{\mathrm{for}\;n_{IT}\;\mathrm{IT}\;\mathrm{SSNs}}{\underset{︸}{\sum_{m=1}^{n_{IT}}\Xi_{m}\left(p_{m}^{C}\left(t\right)\right)}}+\underset{\mathrm{for}\;n_{MT}\;\mathrm{MT}\;\mathrm{SSNs}}{\underset{︸}{\sum_{m=n_{IT}+1}^{n_{IT}+n_{MT}}\Xi_{m}\left(p_{m}^{C}\left(t\right)\right)}}-\chi_{b}\left(\tau_{rp}\right)$$

Therefore, similar to Equation (42), $\Delta \vartheta_{b}^{I}\left(\tau_{rp}\right)$ is also approximately formulated as:

(47) $$\Delta \vartheta_{b}^{I}\left(\tau_{rp}\right)\approx \sum_{m\in N}\log_{2}\left(\frac{p_{m}\left(t\right)}{p_{m}^{C}\left(t\right)}\right)$$

**Proposition** **6.** *Under the condition of the transmit power* $p_{m}\left(t\right)=P_{m}^{\max}$ *for the m-th SSN,* $\Delta \vartheta_{b}^{I}\left(\tau_{rp}\right)$ *satisfies the following upper bound:*

(48) $$\Delta \vartheta_{b}^{I}\left(\tau_{rp}\right)\leq \sum_{m\in N}\log_{2}\left(1/\left(1-\frac{H_{m}\left({ϒ}_{d}\left(m\right)\|{ϒ}_{f}\left(m\right)\right)\tau_{rp}}{2\left(r+1\right)\sum_{m\in N}e^{\delta_{i}\cdot D_{m}\left(P_{d}\|P_{f}\right)}}\right)\right)$$

**Proof.**  Similar to the proof of Proposition 5, it is easy to identify the upper bound in Equation (48), thus completing the proof. ☐

## 6. Simulation Results

**Figure 5:**
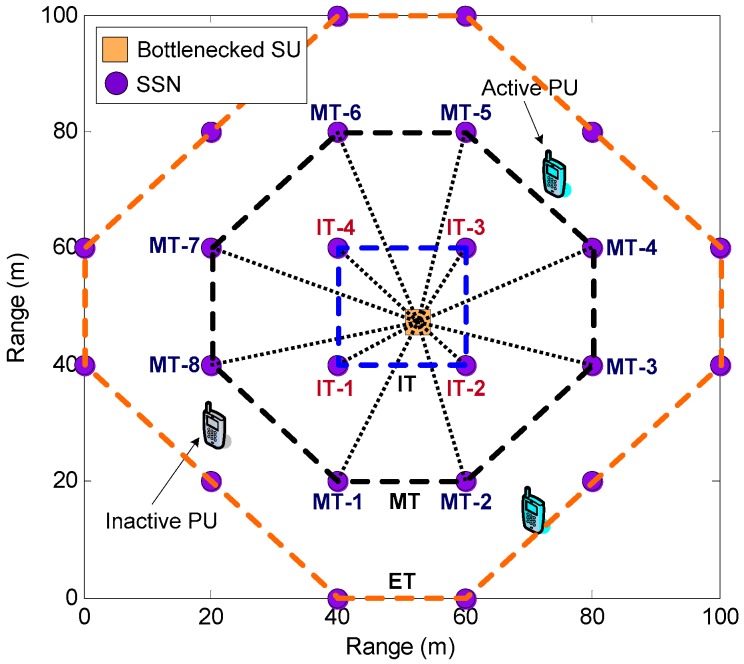
Simulation scenario: the rectangular grid topology with three-tier structure within a torus area of 100 m × 100 m. The dotted lines correspond to the transmissions of the spectrum sensing results from interior tier (IT) and middle tier (MT) spectrum sensor nodes to bottlenecked secondary user. SU: secondary user; PU: primary user; ET: exterior tier.

In this section, we provide simulation results to evaluate the performance of the proposed congestion mitigation approach by using the distributed power control framework for IT and MT SSNs in the rectangular grid based SN-CRN, and investigate the impact of key system parameters on the performance. Particularly, our simulations pay more attention to evaluate the effect of the proposed distributed power control framework for IT and MT SSNs on the reduction of the amount of the data sequence of SSR in the internal buffer of bottlenecked SU *b*. Technically, the reduction of the amount of the data sequence of SSR in the internal buffer will free the buffer capacity of bottlenecked SU *b*, which can bring about the congestion mitigation for bottlenecked SU *b*. As illustrated in Figure 5, all the simulations are carried out on a rectangular grid topology within a torus area of 100 m × 100 m where one bottlenecked SU is randomly placed within the area of IT over three-tier structure. It suffices to mention that the horizontal or vertical distance between any SSNs is initialized to be d = 20 m. For convenience, the IT SSN and the MT SSN are marked by IT-*m* and MT-*n* in sequence for $m\in N_{IT}$ and $n\in N_{MT}$, respectively, as shown in Figure 5. We assume that there are $N_{c}$ = 12 licensed uplink channels allocated to PUs. The SOP usage probability $\delta_{b}$ is set to 0.65 for the bottlenecked SU. Also, the total average power constraint assigned to IT and MT SSNs are assumed to $P_{av}^{IT}$ = 18 dBm and $P_{av}^{MT}$ = 8 dBm, respectively, to mitigate the interference among IT and MT SSNs. As for the AWGN multiple-access channel, the path-loss exponent κ has been set to 8, and the channel bandwidth used for SSNs is assumed to be 2 MHz according to IEEE 802.15.4a channel model. The constant K under the channel capacity formulation given in Equation (4) is defined as 0.005. In addition, the noise power spectral density $N_{0}$ under this channel model is initialized as −3 dBm. We also adopt the receiving reference power by bottleneck SU *b* $P_{0}$ = 20 dBm under the reference distance $d_{0}$ = 20 m.

**Figure 6:**
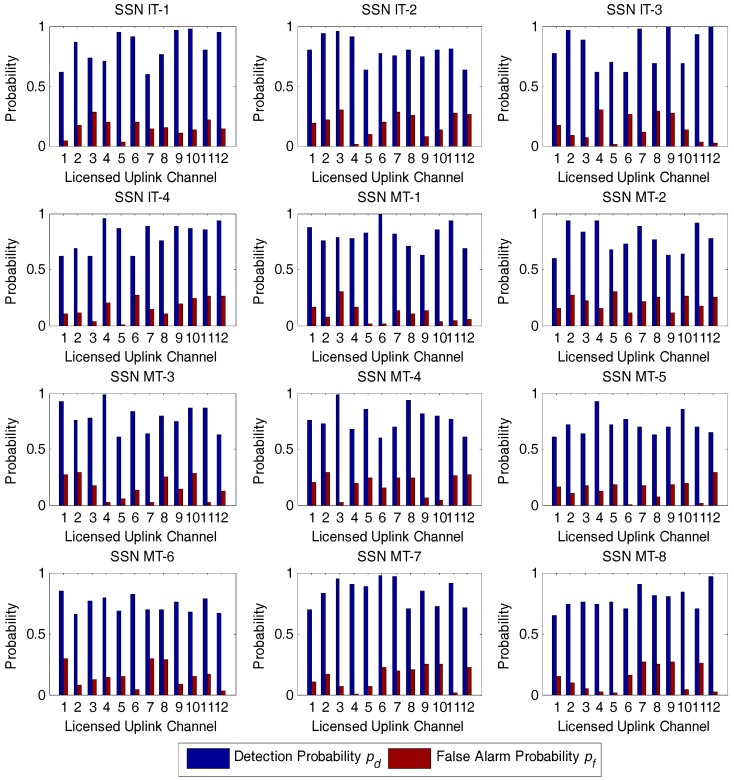
The detection and false alarm probabilities for spectrum sensor nodes from interior tier and middle tier over 12 uplink channels. IT: interior tier; MT: middle tier; SSN: spectrum sensor node.

In all the simulations, the detection and false alarm probabilities for IT and MT SSNs over $N_{c}=12$ licensed uplink channels have been initialized as in Figure 6. We also set the number of subintervals in Algorithm 1 to be the same value for all SSNs, i.e., ξ=ς=8. We also assume that the minimum value $X_{d}$ and the maximum value $Y_{d}$ in derivation of the detection probability distribution are set to 0.6 and 1, respectively. Likewise, the minimum value $X_{f}$ and the maximum value $Y_{f}$ in derivation of the false alarm probability distribution are set at 0 and 0.3, respectively.

**Figure 7:**
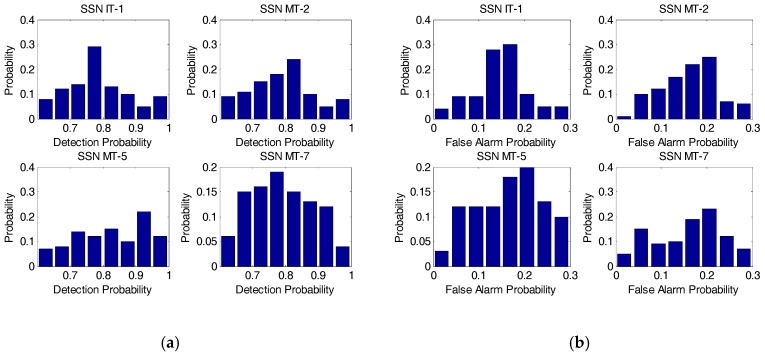
The detection probability and false alarm probability distributions of the selected spectrum sensor nodes involving IT-1, MT-2, MT-5, and MT-7. (**a**) Detection probability distribution; (**b**) False alarm probability distribution. IT: interior tier; MT: middle tier; SSN: spectrum sensor node.

For simplicity, we deliberately choose some of the IT and MT SSNs in the simulation including IT-1, MT-2, MT-5, and MT-7, to evaluate the performance of our developed approach. Specifically, the detection probability and false alarm probability distributions of the selected SSNs from IT and MT generated from Algorithm 1 are assumed to comply with the corresponding distributions as given by Figure 7.

The proposed OTPA algorithm for both the NoCoPC problem and the CoPC problem under the distributed power control framework for IT and MT SSNs is compared with the well-known power balancing (PB) algorithm in [35]. The PB algorithm is also a SNR balancing constrained power control iterative method which iteratively searches for decentralized transmit power level updated from the *l*-th iteration to the (*l* + 1)-th iteration. Let $\gamma_{m}^{tar}$ denote the target SNR for the *m*-th SSN to maintain a certain QoS requirement, for m∈N. In this simulation, the target SNR can be set to $\gamma_{m}^{tar}=7$ dB. Therefore, the PB algorithm iteratively updates the transmit power for the *m*-th SSN according to [35]:

(49) $$p_{m}^{\left(l+1\right)}=\min\left\{P_{m}^{\max},\left(\frac{\gamma_{m}^{tar}}{\gamma_{m}^{\left(l\right)}}\right)\cdot p_{m}^{\left(l\right)}\right\}$$

**Figure 8:**
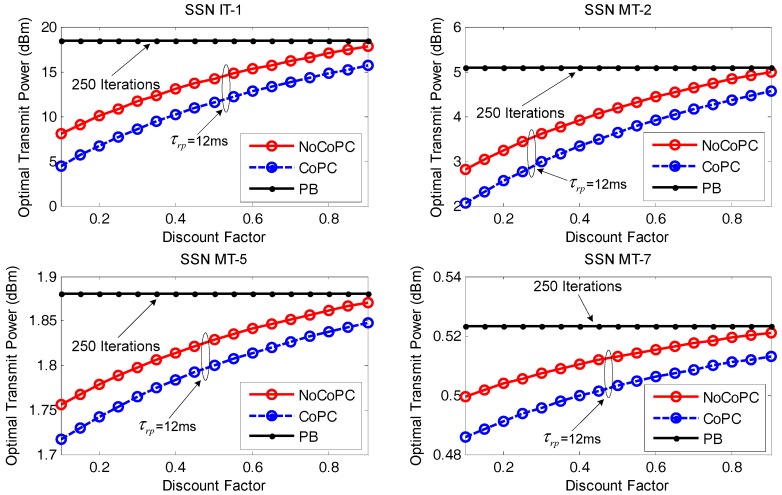
Optimal transmit power vs. discount factor *r* ($\tau_{rp}=12$ ms, *l* = 250 iterations). NoCoPC: noncooperative power control; CoPC: cooperative power control; PB: power balancing.

First the optimal transmit power is compared between PB algorithm with 250 iterations and our proposed distributed power control framework via the noncooperation and cooperation scenarios under the slot $\tau_{rp}$ = 12 ms with the evolution of discount factor *r*, as exhibited in Figure 8. It is shown that an increased discount factor from 0.1 to 0.9 will enhance the optimal transmit power of selected SSNs (i.e., IT-1, MT-2, MT-5, and MT-7) under the proposed OTPA algorithm for both the NoCoPC problem and the CoPC problem within the slot $\tau_{rp}$ = 12 ms. This is due to the fact that the discount factor *r* has an affect on the optimal transmit power of selected SSNs via Equations (28) and (34). Specifically, the optimal transmit power of selected SSNs grows in proportion to discount factor *r*. It is also revealed that the optimal transmit power of selected SSNs under a given discount factor satisfy the average power constraint of $P_{av}^{IT}$ = 18 dBm and $P_{av}^{MT}$ = 8 dBm. Thus, the optimal transmit power of selected SSNs will not be adjusted through OTPA algorithm. Comparing the performance of the NoCoPC problem and CoPC problem with the slot $\tau_{rp}$ = 12 ms, we can also observe that the optimal transmit power of selected SSNs by using PB algorithm presents a fixed constant value. The reason for this is that the optimal transmit power of the selected SSN by using PB algorithm converges to an expected equilibrium point after 250 iterations. It is also interesting that Proposition 2 has turned out that the optimal transmit power of selected SSNs derived by NoCoPC problem converges to a fixed Nash equilibrium point. Moreover, Proposition 4 has also guaranteed that the optimal transmit power of selected SSNs under the CoPC problem will reach to a fixed value. From Figure 8, it is implicitly revealed that the optimal transmit power of selected SSNs by the OTPA algorithm is obviously lower than that of PB algorithm. This observation is reasonable since PB algorithm generates more power consumption to maintain the target SNR. However, the optimal transmit power of selected SSNs via the proposed OTPA algorithm fully depends on the maximum transmit power of the selected SSNs and the pricing factors in differential game model.

**Figure 9:**
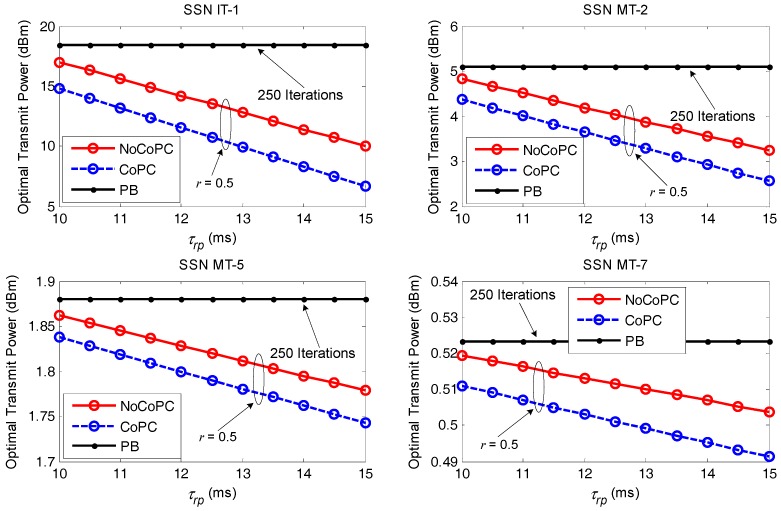
Optimal transmit power vs. the slot $\tau_{rp}$ (*r* = 0.5, *l* = 250 iterations). NoCoPC: noncooperative power control; CoPC: cooperative power control; PB: power balancing.

Figure 9 displays the optimal transmit power comparison between PB algorithm with 250 iterations and our proposed distributed power control framework under discount factor r = 0.5 with the evolution of the slot $\tau_{rp}$ from 10 ms to 15 ms. As can be seen, the proposed OTPA algorithm for both the NoCoPC problem and CoPC problem under the condition of discount factor r = 0.5 outperforms PB algorithm with 250 iterations significantly in terms of the optimal transmit power of selected SSNs with the growth of the slot $\tau_{rp}$. This result further validates PB algorithm will result in much more power consumption aiming to maintain the target SNR. However, the optimal transmit power of selected SSNs by using the proposed OTPA algorithm entirely depends upon maximum transmit power of selected SSNs and pricing factors in differential game model. We can also observe that the optimal transmit power of selected SSNs of the proposed OTPA algorithm for the CoPC problem under discount factor r = 0.5 is considerably lower than that of the NoCoPC problem. That is, the proposed OTPA algorithm for the CoPC problem outperforms that of the NoCoPC problem. This can be intuitively explained by the fact that there exists an operation of summing with respect to $e^{\delta_{i}\cdot D_{m}\left(P_{d}\|P_{f}\right)}$ in the denominator of the analytical cooperative optimal transmit power $p_{m}^{C}\left(t\right)$. Moreover, the increase of the slot $\tau_{rp}$ will generate lower transmit power for both the NoCoPC problem and CoPC problem with discount factor r = 0.5. This is because based on Equations (28) and (34), the optimal transmit power for both the NoCoPC problem and CoPC problem is inversely proportional to the slot $\tau_{rp}$. This observation emphasizes the importance of selecting the proper time interval of slot $\tau_{rp}$ on the optimal transmit power.

**Figure 10:**
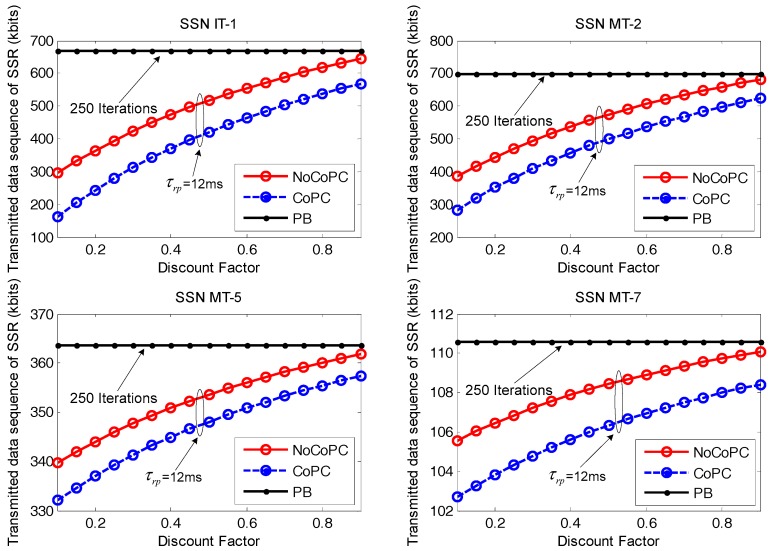
Transmitted data sequence of SSR vs. discount factor *r* ($\tau_{rp}=12$ ms, *l* = 250 iterations). NoCoPC: noncooperative power control; CoPC: cooperative power control; PB: power balancing.

In Figure 10, we look at the performance of the transmitted data sequence of SSR from selected SSNs to bottlenecked SU *b* during the slot $\tau_{rp}$ = 12 ms with the evolution of discount factor *r* from 0.1 to 0.9. From the results, we can see the transmitted data sequence of SSR for selected SSNs gradually increase with the growth of discount factor *r*. Meanwhile, the transmitted data sequence of SSR for PB algorithm with 250 iterations and our proposed distributed power control framework under the condition of the slot $\tau_{rp}$ = 12 ms tends to be close when discount factor *r* = 0.9. The reason for this is that the transmitted data sequence of SSR can be approximately expressed as a function of channel capacity according to Equation (38), which is in direct proportion to the optimal transmit power of selected SSNs. It has also been shown that an increased discount factor from 0.1 to 0.9 will increase the optimal transmit power of selected SSNs under the proposed OTPA algorithm for both the NoCoPC problem and CoPC problem with the slot $\tau_{rp}$ = 12 ms. Consequently, higher optimal transmit power yields more transmitted data sequence of SSR.

**Figure 11:**
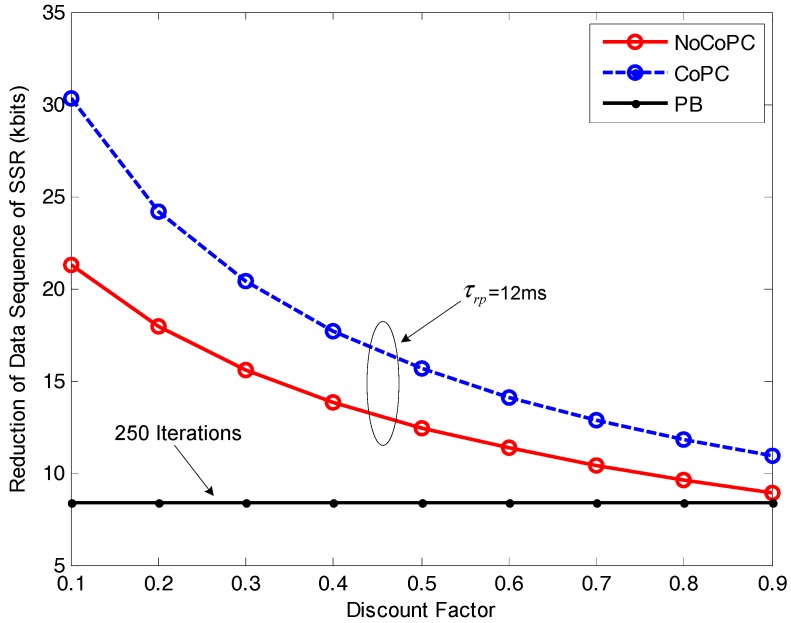
Reduction of data sequence of spectrum sensing results for of selected spectrum sensor nodes vs. discount factor *r* ($\tau_{rp}=12$ ms, *l* = 250 iterations). NoCoPC: noncooperative power control; CoPC: cooperative power control; PB: power balancing.

In Figure 11, we examine the impact of discount factor *r* from 0.1 to 0.9 on the reduction of data sequence of SSR with respect to selected SSNs in the internal buffer of bottleneck SU *b*. It can be observed from the figure that the reduction of data sequence of SSR by our proposed congestion mitigation approach under the slot $\tau_{rp}$ = 12 ms will gradually decrease with the growth of discount factor *r*, except that the reduction result by PB algorithm with 250 iterations appears to a fixed constant value. This trend is the result of the inverse relationship between the reduction of the data sequence of SSR $\Delta \vartheta_{b}^{I}\left(\tau_{rp}\right)$ and the optimal transmit power of selected SSNs $p_{m}^{NC}\left(t\right)$ for the NoCoPC problem or $p_{m}^{C}\left(t\right)$ for the CoPC problem according to Equations (42) and (47). Additionally, in Figure 11, we can also observe that the proposed congestion mitigation approach by using the OTPA algorithm for the CoPC problem with the slot $\tau_{rp}$ = 12 ms achieves the higher reduction of the data sequence of SSR compared with those of the NoCoPC problem and PB algorithm with 250 iterations. In other words, through cooperation among all IT and MT SSNs, the reduction of the data sequence of SSR in the internal buffer of bottlenecked SU *b* can be further improved. This observation has verified the analytical derivation of the proposed congestion mitigation approach. The explanation is twofold: (i) The proposed OTPA algorithm for the CoPC problem outperforms that of the NoCoPC problem owing to the lower cooperative optimal transmit power $p_{m}^{C}\left(t\right)$ obtained by the CoPC problem compared with the noncooperative optimal transmit power $p_{m}^{NC}\left(t\right)$ by the NoCoPC problem; (ii) The reduction of the data sequence of SSR is inversely proportional to the optimal transmit power of selected SSNs.

**Figure 12:**
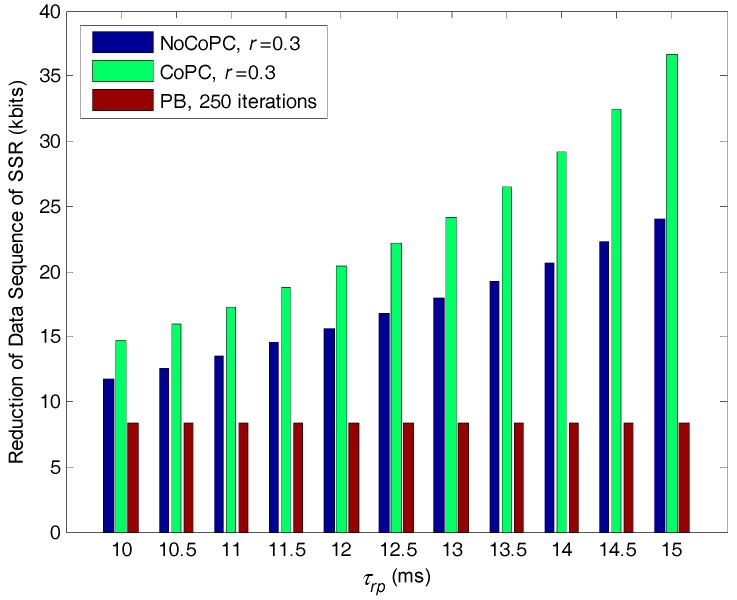
Reduction of data sequence of spectrum sensing resultsfor of selected spectrum sensor nodes vs. the slot $\tau_{rp}$ (*r* = 0.3, *l* = 250 iterations). NoCoPC: noncooperative power control; CoPC: cooperative power control; PB: power balancing.

Figure 12 shows the comparison of the reduction of data sequence of SSR with respect to selected SSNs in the internal buffer of bottlenecked SU *b*, versus the slot $\tau_{rp}$ under the condition of discount factor *r* = 0.3, for the proposed congestion mitigation approach and PB algorithm with 250 iterations. As seen from Figure 11, the reduction of data sequence of SSR through PB algorithm has converged to the same constant value after the 250 iterations. This is the direct influence that the optimal transmit power of selected SSN will converge to an expected equilibrium point by using PB algorithm. As expected, the reduction of data sequence of SSR by the proposed congestion mitigation approach with discount factor *r* = 0.3 is larger than that of PB algorithm with 250 iterations. This is because our proposed approach can obtain smaller optimal transmit power than PB algorithm. According to Equations (42) and (47), the smaller optimal transmit power will lead to the more reduction of data sequence of SSR with respect to selected SSNs. It is also interesting that with the growth of the slot $\tau_{rp}$ the reduction of data sequence of SSR by our proposed approach will gradually increase, and the reduction by the CoPC problem is much higher than that of the NoCoPC problem. This result can be interpreted by the fact that the optimal transmit power of selected SSNs by the CoPC problem under discount factor r = 0.3 is clearly lower than that of the NoCoPC problem with the growth of the slot $\tau_{rp}$. This result further gives rise to the larger reduction of data sequence of SSR.

## 7. Conclusions and Future Work

In this paper, we have developed a congestion mitigation approach by employing the distributed power control framework for SSNs in the rectangular grid based SN-CRN. Particularly, we defined the relative divergence between the detection probability and false alarm probability for a SSN under any uplink channel by adopting a Kullback-Leibler divergence framework. After deriving the detection probability and false alarm probability distributions for SSN according to mathematical statistics, we characterized the stability metric of local spectrum sensing based on entropy modeling framework. Aiming to gain the tradeoff between channel capacity and energy consumption, the distributed power control framework for IT and MT SSNs was proposed, and the power control problem was formulated as differential game model by taking into account the utility function maximization with linear differential equation constraint in regard to energy consumption. Further, we derived the theoretic optimal solutions to this game model under the scenario of cooperation or noncooperation via dynamic programming. Based on the obtained optimal transmit power of SSNs, we devised the congestion mitigation approach for bottleneck SU by alleviating buffer load over its internal buffer, and validated its performance with simulations.

What we have discussed in this paper is the portion of the foundation for SN-CRN. Possible directions for future work within this research involve examining the effect of imperfect CSI and outage constraint on distributed power control for SSNs by formulating the uncertain relation between the wireless channel conditions and the corresponding estimates. As another future work, we will try to investigate congestion mitigation approaches in future 5G mobile systems with the novel network architecture and networking technologies [36], e.g., fog computing-based radio access networks and network slicing-based mobile networks in currently practical applications.

## Appendix A

**Proof of Proposition** **1.** For mathematical tractability, the problem P1 can be converted into an finite horizon differential game model by relaxing the terminal time $t_{0}^{\prime }+\tau_{rp}$ to explore when $t_{0}^{\prime }+\tau_{rp}$ approaches ∞ (i.e., $t_{0}^{\prime }+\tau_{rp}\to \infty$) as infinite time horizon. Under this conversion, we then perform the maximization operation of the right hand side of Equation (25) with respect to $p_{m}\left(t\right)$. After some simplifications, we have the following expression of the noncooperative optimal solution:

(A1) $$p_{m}^{NC}\left(t\right)=P_{m}^{\max}+\frac{\partial V_{m}^{NC}\left(p_{m},E_{m}\right)}{\partial E_{m}}\frac{P_{m}^{\max}H_{m}\left({ϒ}_{d}\left(m\right)\|{ϒ}_{f}\left(m\right)\right)\tau_{rp}}{2}$$

By substituting $p_{m}^{NC}\left(t\right)$ into Equation (25), after some algebraic manipulations, we have:

(A2) $$\begin{array}{l} \;rV_{m}^{NC}\left(p_{m},E_{m}\right)-\frac{P_{m}^{\max}H_{m}\left({ϒ}_{d}\left(m\right)\|{ϒ}_{f}\left(m\right)\right)\tau_{rp}^{2}}{4}{\left(\frac{\partial V_{m}^{NC}\left(p_{m},E_{m}\right)}{\partial E_{m}}\right)}^{2}+{℘}_{m}^{C}E_{m}\left(t\right)\\ =\frac{\partial V_{m}^{NC}\left(p_{m},E_{m}\right)}{\partial E_{m}}\left(E_{A}\left(t\right)-\tau_{rp}\left(P_{m}^{\max}+\frac{\partial V_{m}^{NC}\left(p_{m},E_{m}\right)}{\partial E_{m}}\frac{P_{m}^{\max}H_{m}\left({ϒ}_{d}\left(m\right)\|{ϒ}_{f}\left(m\right)\right)\tau_{rp}}{2}\right)-E_{m}\left(t\right)\right) \end{array}$$

To proceed, we derive the derivative of $V_{m}^{NC}\left(p_{m},E_{m}\right)$ with respect to $E_{m}\left(t\right)$ in Equation (A2). Upon solving the partial differential equation, after some simplifications, we now can express the function $V_{m}^{NC}\left(p_{m},E_{m}\right)$ as the partial differential equation constraint given by:

(A3) $$\frac{\partial V_{m}^{NC}\left(p_{m},E_{m}\right)}{\partial E_{m}}=-\frac{{℘}_{m}^{C}}{r+1}$$

This completes the proof. ☐

## Appendix B

**Proof of Proposition** **3.** The proof is similar to Proposition 1. The only difference is that the objective function of the cooperative power control problem is to maximize the sum of the utility functions of all players. For convenience of derivation, we also relax the time interval of the game and discuss the infinite-horizon differential game (i.e., $\tau_{rp}\to \infty$), and we also set $t_{0}^{\prime }=0$. By performing the maximization operation of the right hand side of Equation (31) with respect to the transmit power of all players $p_{1}\left(t\right),p_{2}\left(t\right),\cdots ,p_{\left|N\right|}\left(t\right)$, after some simplifications, we have:

(A4) $$p_{m}^{C}\left(t\right)=P_{m}^{\max}+\frac{\partial W_{m}^{C}\left(p_{m},E_{m}\right)}{\partial E_{m}}\frac{P_{m}^{\max}H_{m}\left({ϒ}_{d}\left(m\right)\|{ϒ}_{f}\left(m\right)\right)\tau_{rp}}{2}$$

By substituting $p_{m}^{C}\left(t\right)$ into Equation (31), after some algebraic manipulations, we obtain:

(A5) $$\begin{array}{l} \;W_{m}^{C}\left(p_{m},E_{m}\right)-\sum_{m\in N}\left(\frac{P_{m}^{\max}H_{m}\left({ϒ}_{d}\left(m\right)\|{ϒ}_{f}\left(m\right)\right)\tau_{rp}^{2}}{4r}{\left(\frac{\partial W_{m}^{C}\left(p_{m},E_{m}\right)}{\partial E_{m}}\right)}^{2}-\frac{{℘}_{m}^{C}E_{m}\left(t\right)}{r}\right)\\ =\frac{\partial W_{m}^{C}\left(p_{m},E_{m}\right)}{\partial E_{m}}\left(\frac{E_{A}\left(t\right)}{r}-\frac{\tau_{rp}}{r}\left(P_{m}^{\max}+\frac{\partial W_{m}^{C}\left(p_{m},E_{m}\right)}{\partial E_{m}}\frac{P_{m}^{\max}H_{m}\left({ϒ}_{d}\left(m\right)\|{ϒ}_{f}\left(m\right)\right)\tau_{rp}}{2}\right)-\frac{E_{m}\left(t\right)}{r}\right) \end{array}$$

Next, we try to derive the derivative of $W_{m}^{C}\left(p_{m},E_{m}\right)$ with respect to $E_{m}\left(t\right)$ in Equation (A5). Upon solving the partial differential equation, after some basic mathematical manipulations, we obtain the final result in Equation (32), thus completing the proof. ☐
